# mTORC2: The other mTOR in autophagy regulation

**DOI:** 10.1111/acel.13431

**Published:** 2021-07-12

**Authors:** Josué Ballesteros‐Álvarez, Julie K. Andersen

**Affiliations:** ^1^ Buck Institute for Research on Aging Novato California USA

**Keywords:** AKT, autophagy, FOXOs, mTORC2, SGK‐1

## Abstract

The mechanistic target of rapamycin (mTOR) has gathered significant attention as a ubiquitously expressed multimeric kinase with key implications for cell growth, proliferation, and survival. This kinase forms the central core of two distinct complexes, mTORC1 and mTORC2, which share the ability of integrating environmental, nutritional, and hormonal cues but which regulate separate molecular pathways that result in different cellular responses. Particularly, mTORC1 has been described as a major negative regulator of endosomal biogenesis and autophagy, a catabolic process that degrades intracellular components and organelles within the lysosomes and is thought to play a key role in human health and disease. In contrast, the role of mTORC2 in the regulation of autophagy has been considerably less studied despite mounting evidence this complex may regulate autophagy in a different and perhaps complementary manner to that of mTORC1. Genetic ablation of unique subunits is currently being utilized to study the differential effects of the two mTOR complexes. RICTOR is the best‐described subunit specific to mTORC2 and as such has become a useful tool for investigating the specific actions of this complex. The development of complex‐specific inhibitors for mTORC2 is also an area of intense interest. Studies to date have demonstrated that mTORC1/2 complexes each signal to a variety of exclusive downstream molecules with distinct biological roles. Pinpointing the particular effects of these downstream effectors is crucial toward the development of novel therapies aimed at accurately modulating autophagy in the context of human aging and disease.

## Graphical abstract

mTORC2 is a negative regulator of macroautophagy and chaperone‐mediated autophagy via its ability to signal to various downstream effectors shown to modulate autophagy in mammals including AKT, PKC, SGK‐1, and FOXO transcription factors. In this review, we highlight the importance of identifying novel mechanisms that target mTORC2 specifically but also of dissecting the role of the downstream effectors through substrate‐specific interactions.

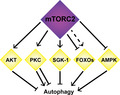

**Abbreviations**
4EBP1: Eukaryotic translation initiation factor 4E‐binding protein‐1
AMP: Adenosine monophosphate
AMPK: AMP‐activated protein kinase
ARG2: Arginase 2
ATG: Autophagy‐related gene
ATP: Adenosine triphosphate
BCL2: B‐cell lymphoma 2
BNIP3: BCL2/adenovirus E1B 19 kDa protein‐interacting protein 3
C.: Caenorhabditis
cAMP: Cyclic AMP
CDK2: Cell division protein kinase 2
CLEAR: Coordinated Lysosomal Expression and Regulation
CMA: Chaperone‐mediated autophagy
CREB: cAMP‐response element‐binding protein
CRIM: Conserved region in the middle
D.: Drosophila
Daw: Dawdle
DEP: Disheveled, egl‐10, and pleckstrin
DEPTOR: DEP domain‐containing mTOR‐interacting protein
EGFR: Epidermal growth factor receptor
ERK: Extracellular signal‐regulated kinase
ESCRT: Endosomal sorting complexes required for transport
FKBP12: FK506‐binding protein 12
FNIP2: Folliculin Interacting Protein 2
FOXO: Forkhead box class O
GDP: Guanosine diphosphate
GSK3β: Glycogen synthase kinase 3 beta
GTP: Guanosine‐5'‐triphosphate
HDAC: Histone deacetylase
Hlh‐30: Helix loop helix 30
HSC: Hematopoietic stem cell
HSC70: Heat shock cognate 71 kDa
IGF: Insulin growth factor
IKK: IκB kinase
JNK: c‐Jun N‐terminal kinases
LAMP1: Lysosomal‐associated membrane protein 1
LC3A/B: Microtubule‐associated proteins 1A/1B light chain 3A/B
Lgg‐1: LC3, GABARAP and GATE‐16 family 1
LSD: Lysosomal storage disease
MAPK: Mitogen‐activated protein kinase
MEK: Mitogen‐activated protein kinase kinase
MHC: Major histocompatibility complex
MiT/TFE: Microphthalmia/transcription factor E
MITF: Microphthalmia‐associated transcription factor
mLST8: mTOR‐associated protein, LST8 homolog
mSIN1: Mammalian stress‐activated MAP kinase‐interacting protein‐1
mTORC: Mechanistic target of rapamycin complex
NDRG1: N‐Myc Downstream Regulated 1
NRF2: Nuclear factor erythroid 2‐related factor 2
PDGF: Platelet‐derived growth factor
PDK1: Phosphoinositide‐dependent kinase 1
PH: Pleckstrin homology
PHLPP1: PH domain and leucine‐rich repeat protein phosphatase 1
PI3K: Phosphoinositide 3‐kinase
PIF: PDK1 interacting fragment
PIKK: Phosphatidylinositol 3‐kinase‐related kinase
PIP3: Phosphatidylinositol (3,4,5)‐trisphosphate
PKB: Protein kinase B
PKC: Protein kinase C
PRAS40: 40‐kDa proline‐rich Akt substrate
RAB: Ras‐related protein
RAPTOR: Regulatory‐associated protein of mTOR
Rb: Retinoblastoma
RHEB: Ras homolog enriched in brain
RICTOR: Rapamycin‐insensitive companion of mammalian target of rapamycin
RTK: Receptor tyrosine kinase
*S*.: Saccharomyces
S6K1: Ribosomal protein S6 kinase beta‐1
Ser: Serine
SGK: Serum and glucocorticoid‐regulated kinase
ShRNA: Short hairpin RNA
SIRT: Sirtuin
Skn‐1: Skinhead 1
SNARE: Soluble N‐ethylmaleimide‐sensitive factor attachment protein receptor
TFE3: Transcription factor E3
TFEB: Transcription factor EB
Thr: Threonine
TSC: Tuberous sclerosis complex
ULK1: Unc‐51 like autophagy activating kinase 1
vATPase: Vacuolar ATPase
VDAC1: Voltage‐dependent anion channel 1
Wnt: Wingless and Int‐1
ZKSCAN3: Zinc finger with KRAB and SCAN domains 3

## 1 THE ENDOSOMAL SYSTEM

Endosomes are membrane‐bound compartments contained within eukaryotic cells. These vesicles originate from the trans‐Golgi and can be catalogued as early endosomes, recycling endosomes, late endosomes, or fully mature lysosomes. Endosomes store and sort intracellular material that is subsequently recycled back to the plasma membrane or to the Golgi, whereas acidified late endosomes mature into lysosomes. Fully mature acidic lysosomes are capable of fusing with autophagosomes, targeting their content for protein degradation in a catabolic process known as autophagy which is vital for intracellular recycling and clearance of cellular debris. The nature of the material that is engulfed by autophagosomes is varied and includes misfolded protein aggregates, oxidized lipids, pathogens, and damaged intracellular organelles such as mitochondria which can have deleterious effects on cellular health if not properly cleared. The products from endocytic vesicles or autophagic degradation are subsequently recycled to meet the nutritional needs of the cell for energy production or as building blocks for the biosynthesis of new cellular components.

Autophagy can be roughly divided into three different subtypes in mammalian cells. Microautophagy features the formation of small invaginations in the lysosomal membrane that engulf cytosolic cargo through complex fusion/fission membrane dynamics that are yet to be fully elucidated but are likely to be dependent on endosomal sorting complexes required for transport (ESCRT) proteins and/or soluble N‐ethylmaleimide‐sensitive factor attachment protein receptor (SNARE) proteins (Schuck, 2020).

Macroautophagy, to date the most well‐studied autophagy subtype, involves the fusion of the autophagosome with an acidic lysosome, forming an autolysosome. In turn, macroautophagy can be divided into bulk autophagy or selective autophagy, the latter referring to the degradation of specific subcellular structures, such as mitophagy of mitochondria, lipophagy of lipid droplets, or chlorophagy of chloroplasts. At least 35 autophagy‐related genes (ATGs) have been identified so far, with a subset (the “core” ATGs) being conserved across eukaryotic organisms from yeast to mammals (Bento et al., 2016; Mizushima & Komatsu, 2011). These govern processes including autophagosome induction and formation, expansion, closure, and fusion with lysosomes.

The third subtype of autophagy described in mammals, namely chaperone‐mediated autophagy (CMA), consists of the specific targeting of a substrate for degradation by the formation of a complex with heat shock cognate 71 kDa (HSC70) proteins. The complex is subsequently transported to the lysosomal membrane where it binds a CMA receptor and is internalized. CMA is unique in that, following receptor recognition, substrate proteins traverse the membrane rather than being engulfed by nascent endosomes (Kaushik & Cuervo, 2012).

Pathways involved in endosomal trafficking include the fusion of mature lysosomes with vesicles that contain extracellular material that has been taken up through the process of endocytosis for further digestion. Additionally, a small fraction of cellular lysosomes is secretory lysosomes, also called lysosome‐related organelles. Secretory lysosomes localize closer to the plasma membrane. Here, exocytosis has been shown to be calcium‐dependent and regulated by the transcription factor EB (TFEB), which controls the transcription of genes involved in translocation to the plasma membrane and unloading of cellular metabolites (Medina et al., 2011). Lysosomal exocytosis is very active in certain cell lineages: melanocytes that secrete melanosomes filled with melanin pigment (Raposo & Marks, 2002), osteoclasts that resorb bone material by endocytosis and transport the vesicles through the cytoplasm to the opposite pole at the basal plasma membrane, releasing their content into the extracellular space (Salo et al., 1997), and spermatozoa that secrete hydrolases during fertilization (Tulsiani et al., 1998). Lysosomal exocytosis is important for the immune function through the secretion of antigen‐loaded major histocompatibility class II complexes (MHC‐II) by dendritic cells and for cytotoxic T‐cell secretory degranulation (Pu et al., 2016). Lysosomes also have been shown to migrate to the plasma membrane to promote repair, a process dysregulated in muscular dystrophy (Han et al., 2007).

The endosomal‐lysosomal network has traditionally been considered as merely a mechanism for intracellular sorting and delivery, degradation, and recycling. However, an ever‐increasing number of studies highlight a complex role for endosomal vesicles and lysosomes as major signaling hubs that link environmental cues such as amino acid availability through multiple signaling pathways to gene regulation involved in cellular metabolism (Dibble & Manning, 2013). The modulation of mitogenic signals through degradation of epidermal growth factor receptor (EGFR) and other receptor tyrosine kinases (RTKs) (Burke et al., 2001) are examples of these.

### 1.1 Autophagy and endosomal traffiking in human disease and aging

Defective lysosomal function is the cause of a group of more than 50 rare inherited metabolic disorders designated as lysosomal storage diseases (LSDs). These disorders are characterized by mutations that usually result in deficiency of a single enzyme required for the metabolism of substrates, leading to enlarged lysosomal vacuoles filled with undegraded material often localized adjacent to the nucleus. They are further classified, depending on the substrate that accumulates into lipid storage disorders, mucopolysaccharidoses, or glycoprotein storage disorders. Pompe disease, for example, is a LSD characterized by an accumulation of glycogen in the lysosomes of muscle cells, nervous system, and liver. To date, there are no cures for LSDs and treatments are mostly symptomatic.

Recently, increasing attention has been given to a potential link between abnormal lysosomal function and neurodegeneration and the induction of autophagy as a potential therapeutic target for conditions such as Huntington's disease, Parkinson's disease, and Alzheimer's disease, where pathogenic protein aggregation is a shared characteristic hallmark. These conditions have been attributed to changes in, among others, mTORC1, dynein, and Ras‐related protein‐7 (RAB7) activity (Caviston et al., 2011; Erie et al., 2015; Wen et al., 2017), preventing degradation of protein deposits by the autolysosomal pathway through a mechanism not yet fully elucidated. Accumulation of lysosomes that disturb the normal morphology of neuronal axons due to blockade of lysosomal proteolysis and vesicle transport has been described in Alzheimer's disease (Lee et al., 2011).

Understanding the role of the autophagy response in cancer biology is crucial in the search for novel effective therapeutics for this group of disorders. Anticancer agents have been shown to activate autophagy which can serve as a means for promoting their survival through efficient use of available nutrients, handling of oxidative stress, and limiting DNA damage. Activated autophagy can be detected at the most hypoxic regions of the tumor where there is considerable metabolic stress, conferring a survival advantage. Autophagy inhibition has been shown to improve the performance of anticancer drugs, including the response to chemotherapy in several human tumors and in preclinical mouse models. Inhibition has been shown to enhance the cytotoxic activity of effector T lymphocytes and natural killer cells against tumoral cells (Amaravadi et al., 2011). Human pancreatic tumors and pancreatic cancer cell lines are dependent on elevated basal levels of autophagy compared to normal pancreatic cells (Yang et al., 2011). Consistent with this, autophagy inhibition has been reported to induce apoptosis and to halt pancreatic tumor growth (Marchand et al., 2015). During oncogenic transformation, lysosomes can undergo changes in pH, subcellular localization, or composition; reductions in RAB7 expression promote lysosomal relocation to the cell periphery which has been documented to occur in prostate cancer and associated with increased tumor invasiveness (Steffan et al., 2014). The authors proposed that the mechanism behind this involves the unloading of proteolytic enzymes into the extracellular space that then digest the extracellular matrix. In addition, the endosomal network can incorporate proteins such as the transmembrane type 1 matrix metalloproteinase into the plasma membrane invadopodia, facilitating tumor migration, invasion, and metastasis (Macpherson et al., 2014; Monteiro et al., 2013). Peripheral lysosomal relocation has also been associated with increased expression of integrins in the plasma membrane, enhancing migration, and cell adhesion necessary for the establishment of new tumor populations (Dozynkiewicz et al., 2012). Importantly, tumor cells that undergo the process of autophagy, cease cell division and movement, and enter a dormant state, while maintaining the capacity to regenerate and resume proliferation when proper growth conditions are restored. Tumor regeneration and re‐emergence from latency is a key issue in cancer management (White & DiPaola, 2009).

Paradoxically, a tumor‐suppressive role for autophagy in line with its known role in clearing damaged organelles and cellular components has also been documented. This role is particularly prominent in hepatocellular carcinoma. Mice carrying deletions of Atg5 or Atg7 display an inactivation of autophagy and a build‐up of cellular waste. Defective autophagy leads to increased DNA‐damaging reactive oxygen species production, genomic instability, and generalized inflammation which can promote tumor initiation (Komatsu et al., 2007; Takamura et al., 2011). Monoallelic loss of *Beclin*‐*1*, a gene required for autophagic induction, has been reported in both ovarian and breast cancer (Liang et al., 1999). Beclin‐1 deletions induce chromosome instability and tumor initiation (Mathew et al., 2007). A very common mutagenesis event in a wide variety of human cancers involve mutations that lead to abnormal activity of the PI3K and mTOR pathways. Hyperactive phosphoinositide 3‐kinase (PI3K) inhibits autophagy and simultaneously stimulates uncontrolled cell growth and proliferation, regardless of nutrient and growth signal availability, which eventually leads to extreme metabolic stress and cell death (Jin et al., 2007), perhaps in a delicate balance that is still advantageous to the tumor. Due to its various known effects, autophagy has been referred to as a double‐edged sword in regard to whether it contributes to the overall suppression or promotion of tumorigenesis (White & DiPaola, 2009). Current efforts are directed to understand how pharmacological modulation of autophagy in cancer could lead to the development of novel, effective therapies depending on the particular autophagy context and whether it is activated or suppressed.

Current research on therapies that aim to prolong lifespan shows that the autophagy pathway is one of those implicated during the aging process. Age‐related impairments in autophagy have been reported in the rat liver and studies in model organisms including *Saccharomyces cerevisiae*, *Caenorhabditis elegans*, and *Drosophila melanogaster* demonstrate that deficiency in key autophagy genes (*Atg*s) results in a reduced lifespan (Martinez‐Lopez et al., 2015). In mammals, protein levels of LAMP2A have been shown to decline in aging mice, correlating with decreased CMA activity. Interestingly, inducible liver overexpression of the LAMP2A receptor helped maintaining functional CMA in the murine aged liver (Zhang & Cuervo, 2008). The effects of pharmacological approaches directed to promote longevity including caloric restriction, sirtuin‐1 (SIRT1) activation, mTOR, and insulin growth factor (IGF) inhibition all require autophagy activation (Rubinsztein et al., 2011). However, there are other documented effects of these approaches that are autophagy‐independent. A better understanding of the role that autophagy plays in the complex biological process of aging will therefore be essential in order to reach the goal of boosting cellular rejuvenation and lifespan extension.

## 2 THE mTOR COMPLEX

**FIGURE 1:**
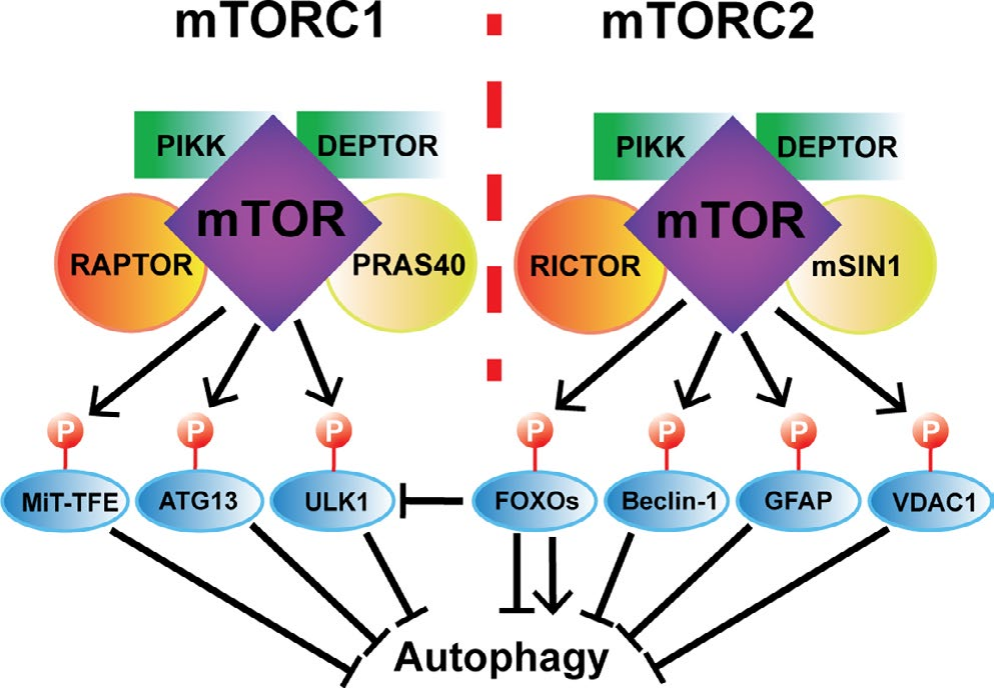
The two distinct mTORC1/2 complexes and their regulation of autophagy. PIKK and DEPTOR are components common to both mTOR complexes, whereas RAPTOR and PRAS40 are specific to mTORC1 and RICTOR and mSIN1 are specific to mTORC2. mTORC1 has been shown to induce phosphorylation of the MiT‐TFE factors, ATG13 and ULK1, which become unable to positively regulate autophagy. In turn, mTORC2 induces the phosphorylation of Beclin‐1, GFAP, and VDAC1, and similarly, prevents them from activating autophagy. mTORC2‐mediated phosphorylation of FOXO proteins modulates their subcellular localization leading to various outcomes regarding autophagy regulation (see Figure 3). See details in text

The mechanistic target of rapamycin complex (mTOR) contains several components including phosphatidylinositol 3‐kinase‐related kinase (PIKK) that serve as the core for two distinct complexes: mTORC1 and mTORC2 (Heitman et al., 1991). These two multi‐subunit complexes associate with distinct sets of proteins and localize to particular subcellular compartments as dimers. The disheveled, egl‐10, and pleckstrin (DEP) and domain‐containing mTOR‐interacting protein (DEPTOR) are a negative regulator of mTOR catalytic activity, common to both complexes (Peterson et al., 2009). Specific to mTORC1 are the regulatory‐associated protein of mTOR (RAPTOR) (Hara et al., 2002) and the 40‐kDa proline‐rich Akt substrate (PRAS40) (Sancak et al., 2007). Rapamycin‐insensitive companion of mTOR (RICTOR) and the mammalian stress‐activated MAP kinase‐interacting protein‐1 (mSIN1) form part of the mTORC2 exclusively (Xie et al., 2018; Zoncu et al., 2011). The mTORC1/2 complexes both are capable of sensing various signaling pathways, growth factors, hormones, redox status and certain amino acids and their derivatives, ensuring the availability of adequate resources vital for the modulation of cell metabolism and survival (Bartolome & Guillen, 2014; Hay & Sonenberg, 2004) (Figure 1).

A key characteristic that has allowed researchers to discern between these two complexes is the binding of the FK506‐binding protein 12 (FKBP12) to the macrolide rapamycin, forming a complex that is capable of directly inhibiting mTORC1 (Brown et al., 1994), but not mTORC2 which has thus been deemed to be insensitive to rapamycin (Jacinto et al., 2004). Under conditions of nutrient restoration following serum deprivation, rapamycin prevents the re‐phosphorylation of ribosomal protein S6 kinase beta‐1 (S6K1) by mTORC1 but not that of Ser473 of AKT by mTORC2 (Jia & Bonifacino, 2019). The C‐terminus of the mTORC2‐specific RICTOR component lies adjacent to the FKBP‐12‐rapamycin‐binding domain of mTOR, obstructing it and therefore rendering it ineffective. Interestingly, removal of the C‐terminus of Avo3, the RICTOR homolog in *S*. *cerevisiae*, generated a FKBP12‐rapamycin‐sensitive complex (Gaubitz et al., 2015). However, chronic rapamycin treatment as opposed to acute exposure has been shown to disrupt mTORC2 activity, affecting glucose metabolism (Arriola Apelo et al., 2016; Lamming et al., 2012; Yu et al., 2019), possibly due to binding of rapamycin‐FKBP12 to the nascent pool of mTOR and thus ablating its assembly into mTORC1/2 complexes (Sarbassov et al., 2006). As predicted, prolonged rapamycin treatment has been reported to impair Akt phosphorylation, the canonical downstream target of phosphorylation of mTORC2 (Sarbassov et al., 2006). The conserved region in the middle (CRIM) of Sin1 is a domain that has been shown to mediate recruitment of AGC kinases to mTORC2 and thus required for their mTORC2‐mediated activation (Tatebe et al., 2017). CRIM‐deficient mutants efficiently ablate phosphorylation of AKT, protein kinase C‐alpha (PKCα), and PKCε while not affecting the mTORC1 target S6K1 (Cameron et al., 2011; Murray & Cameron, 2017). These findings suggest the importance of determining the precise mechanistic action of rapamycin versus other mTOR inhibitors in an effort to differentiate the roles of the two mTORC1/2 complexes and further elucidate their multiple mechanisms of action and potential as therapeutic targets.

### 2.1 The role of the mTORC1 pathway in autophagy

The mTORC1 complex has been shown to be regulated by multiple input signals such as hormones, growth factors, amino acids, and fatty acids. One of the main effects of activation of mTORC1 is the phosphorylation of S6K1 and eukaryotic translation initiation factor 4E‐binding protein‐1 (4EBP1), which facilitates the translation of mRNA and positively controls downstream anabolic processes. The tuberous sclerosis complex (TSC) is the main upstream negative regulator of mTORC1, integrating these upstream signals (Inoki & Guan, 2009; Swiatkowski et al., 2017). It stimulates the GTPase activity of the ras homolog enriched in brain (RHEB) protein that hydrolyzes RHEB‐GTP to RHEB‐GDP, maintaining mTORC1 in a switched‐off state. TSC can be inhibited by the AKT, MAPK, and Wnt pathways, whereas it is activated via phosphorylation by AMP‐activated protein kinase (AMPK) (Dalle Pezze et al., 2016), a kinase that becomes active at low ATP:AMP ratio. An important TSC‐independent mechanism of mTORC1 regulation is its inhibition under conditions of amino acid deprivation, connecting cellular nutritional status to the modulation of cell metabolism. Of note, the particular circulating amino acids responsible for the activation of the complex remain elusive. Arginine and leucine have been shown to be required for mTORC1 activation, however, they are not sufficient (Hara et al., 1998). Importantly, in addition to its role as an activator of the mTORC1 inhibitor TSC, AMPK has been shown to become activated by amino acid sufficiency, and subsequently able to place an inhibitory phosphorylation on Raptor (Gwinn et al., 2008). The concurrent activation of mTORC1 and AMPK under nutrient‐rich conditions may suggest a role for AMPK in maintaining autophagy and metabolic homeostasis and an intricate cross‐regulation of the AMPK‐mTORC1 axis (Dalle Pezze et al., 2016). Recent studies support a two‐step mechanism for the activation of mTORC1 by two distinct groups of amino acids, namely priming and activating amino acids (Dyachok et al., 2016). When amino acids are abundant, a conformational change in Rag‐GTPase heterodimers is induced. These heterodimers interact with Raptor, a member of the mTORC1 complex, which in turn localizes the complex to the surface of late endosomes and lysosomes where it is activated by RHEB‐GTP (Martina & Puertollano, 2013; Sancak et al., 2008). This highlights the notion that the endosome‐lysosomal network is a central hub for intracellular signaling and not just merely as a degradation station (Perera & Zoncu, 2016).

Importantly, upon integration of these multiple environmental and chemical cues, mTORC1 is known to play a pivotal role as a negative regulator of catabolic processes such as autophagy in a transcription‐dependent and independent manner. Under a sufficient supply of nutrients, growth factors, and cellular signaling stimuli, mTORC1 is activated at the lysosomal surface and, through phosphorylation, inhibits proteins that are important for autophagy and lysosomogenesis. These include TFEB (Martina et al., 2012; Roczniak‐Ferguson et al., 2012) and transcription factor E3 (TFE3) (Martina et al., 2014), two important transcriptional regulators of these cellular processes, and Unc‐51 like autophagy activating kinase 1 (ULK1) and ATG13 (Hosokawa et al., 2009), proteins that are directly involved in the formation of autophagosomes (Figure 1).

TFEB contains a number of amino acids that undergo phosphorylation by several different kinases. Serine 142 of TFEB has been shown to be phosphorylated by ERK and mTORC1, the latter which also targets serine 211 of TFEB. This takes place at the outer lysosomal membrane and affects the subcellular localization of the protein. Phosphorylated TFEB at the lysosomal membrane is bound by 14‐3‐3 proteins that mask a nuclear localization signal in the vicinity, thereby retaining TFEB in the cytoplasm (Pena‐Llopis et al., 2011; Roczniak‐Ferguson et al., 2012; Settembre et al., 2012). Similarly, mTORC1 phosphorylates TFE3 and microphthalmia‐associated transcription factor (MITF) and regulates their activity through the same mechanism of cytoplasmic retention (Martina et al., 2014; Martina & Puertollano, 2013). Inactivation of the complex due to lack of nutrients or mitogens switches off the mTORC1 and MAPK/ERK pathways, leading to nuclear translocation of these factors and upregulation of target genes. Unphosphorylated TFEB and TFE3 shuttle to the nucleus, where they bind to Coordinated Lysosomal Expression and Regulation (CLEAR) consensus sequences, whereas nuclear MITF has been shown to activate genes involved in melanogenesis in MNT‐1 melanoma cells (Bentley et al., 1994; Hah et al., 2012). Inactive mTORC1‐dependent shuttling of microphthalmia/transcription factor E (MiT/TFE) proteins to the nucleus establishes a negative feedback loop, turning on expression of genes required for mTORC1 activity including folliculin interacting protein 2 (FNIP2), RagC/D, and vATPase and promoting lysosomogenesis and autophagy thereby increasing amino acid pools (Palmieri et al., 2011; Zhang et al., 2015). Nuclear shuttling of TFEB and subsequent enhancement of its transcriptional activity has been shown to lead to induction of autophagy and clearance of intra‐lysosomal aggregates in a model of neurological Batten disease (Palmieri et al., 2017). Interestingly, mTORC1 can be positively regulated through the MAPK/ERK axis by two different routes, pointing to a crosstalk between these two distinct pathways in the regulation of the CLEAR network. Firstly, ERK1/2 downstream of the BRAF/MAPK pathway can phosphorylate RSK which, in turn, activates mTORC1 in melanoma cells and its downstream targets, promoting increased protein translation, growth, and proliferation (Romeo et al., 2013; Xue et al., 2017). Secondly, MAPK signaling leads to phosphorylation of raptor on proline sites prior to raptor‐mediated scaffolding and recruitment to the lysosomes of active mTORC1 (Carriere et al., 2011).

## 3 mTORC2: THE OTHER mTOR

Significantly less is known about mTORC2 as compared to mTORC1, although it has been shown to respond to growth factors such as insulin and to participate in the regulation of cell metabolism and survival, mostly through AKT/protein kinase B (PKB) as its main downstream effector. AKT/PKB is the best described substrate of mTORC2 phosphorylation, to the extent that a great number of the studies investigating mTORC2 activity refer to AKT as the most common readout of its activity. AKT/PKB is a member of the family of AGC kinases that includes PKC and serum and glucocorticoid‐regulated kinase (SGK). Several of the AGC kinases contain a turn motif adjacent to hydrophobic residues that is subject to phosphorylation by mTORC2 (Dai & Thomson, 2019; Pearce et al., 2010; Polak & Hall, 2006). It is important to take into consideration when studying mTORC2 and its effectors that the phosphorylation of this domain in AGC kinases is only required for maximal activation of the kinase and coincides with downstream phosphorylation of specific substrates (Gaubitz et al., 2016). Multi‐step activation of AKT involves the phosphorylation of Thr308 by phosphoinositide‐dependent kinase 1 (PDK1) and Ser473, one of the true targets of mTOR kinase activity that requires the presence of RICTOR (Sarbassov et al., 2005), others being Thr450, Ser477, and Thr479. Thr450 belongs to the turn motif of AKT has been described as constitutively phosphorylated and as responsible for proper folding of the protein (Ikenoue et al., 2008; Manning & Toker, 2017), increasing stability and reducing ubiquitination (Liao & Hung, 2010; Risso et al., 2015). Ser477 and Thr479 phosphorylation at the C‐terminal tail of AKT has been shown to respond to cell division protein kinase 2 (CDK2)/Cyclin A and also mTORC2, promoting active conformation of the protein and further phosphorylation at other residues such as Ser473 (Liu et al., 2014, 2014).

A variety of upstream inputs have been associated with mTORC2‐mediated phosphorylation of Ser73 of AKT. Insulin‐stimulated PI3K promotes the association of mTORC2 with the ribosome which, in turn, correlated with increased phosphorylation of Ser473 of AKT (Zinzalla et al., 2011). RICTOR is required for insulin‐induced phosphorylation of Ser473 (Kumar et al., 2008). IκB kinase alpha (IKKα) has been shown to associate with mTORC2, stimulating its Ser473 phosphorylation (Dan et al., 2016) in the response to inflammation. A regulatory feedback loop involving mTORC2 and AKT has been described, in which PDK1 activates Akt through phosphorylation of Thr308. This leads to activation of mTORC2 through phosphorylation of Sin1 at Thr86, resulting in further enhanced phosphorylation of Akt by mTORC2 (Yang et al., 2015). Amino acid availability has been shown to induce the phosphorylation of Thr308 of Akt, feeding into this positive regulatory loop. The regulation of mTORC2 activation by amino acids is reminiscent of the well‐described function of mTORC1 as a nutrient sensor. However, the particular amino acids that activate mTORC2 seem to be different to those that activate mTORC1, as shown by the different responses of these complexes to different starvation growth medium in vitro (Tato et al., 2011). Recent findings reveal a cross‐regulatory interaction between mTORC2 and mTORC1 in the context of amino acid availability sensing. mTORC2 inhibition leads to increased glutamine efflux and cysteine uptake through phosphorylation of the cysteine‐glutamate antiporter xCT at Ser26 (Gu et al., 2017). In turn, intracellular cysteine has been shown to induce mTORC1 activity (Yu & Long, 2016; Zhang et al., 2021), as genetic ablation of the antiporter inhibits mTORC1 activity (Daher et al., 2019). Thus, mTORC2 can be considered a multifaceted complex that links broad spatial heterogeneity with a wide variety of upstream activating stimuli and downstream cellular effectors (Knudsen et al., 2020).

### 3.1 The mTORC2‐AKT axis in autophagy

Novel findings link mTORC2 activity with the regulation of the autophagy process. In addition to the plasma membrane, nucleus, mitochondria, and other cytoplasmic structures, mTORC2 has been found to localize to the lysosomes (Berchtold et al., 2012; Ebner et al., 2017; Zinzalla et al., 2011). Approximately 29% of endogenous lysosomal‐associated membrane protein‐1 (LAMP1) co‐localizes with mTORC2, indicating that a subgroup of lysosomes are mTORC2‐positive (Jia & Bonifacino, 2019). At the lysosomal level, mTORC2 and AKT have been shown to regulate CMA machinery recruitment (Arias et al., 2015).

Similar to mTORC1, mTORC2 has been shown to be a negative regulator of autophagy. AKT itself has been reported as an mTORC1‐independent negative regulator of autophagy. AKT can directly phosphorylate Beclin‐1 at Ser234 and Ser295, promoting binding of Beclin‐1 to intermediate filaments and autophagy suppression (Mostowy, 2014; Wang et al., 2012). IGF‐1 can inhibit autophagy via activation of mTORC2 and AKT, linking glucose metabolism as a nutritional cue able to modulate autophagy processes through these proteins (Wang & Gu, 2018). In colorectal cancer cells with constitutively activated mTORC1, mTORC2 appears to regulate the levels of basal autophagy and participate in the maintenance of signaling vesicles and tumor cell survival (Lampada et al., 2017, 2017).

A regulatory feedback loop between starvation‐induced autophagy and mTORC2 has been suggested, as starvation initially triggers reduced levels of phosphorylated Ser473, followed by re‐phosphorylation upon prolonged starvation (Bernard et al., 2014). Along these same lines, chronic autophagy has been shown to increase the activity of mTORC2 via the induction of reactive oxygen species (Bernard et al., 2020).

A very compelling study by Arias and colleagues (Arias et al., 2015) investigated the effects of AKT inhibition on chaperone‐mediated autophagy (CMA) in fibroblasts. Treatment with cell‐permeable AKT inhibitors abrogated the phosphorylation of Ser473 of Akt induced by platelet‐derived growth factor (PDGF) which, in turn, activated basal CMA. In this context, the Pleckstrin homology (PH) domain and leucine‐rich repeat protein phosphatase 1 (PHLPP1) were the key phosphatase counteracting phosphorylation of AKT by mTORC2 (Arias et al., 2015). Whereas mTORC2 activity is reduced during starvation and hence inhibits CMA, it increases upon prolonged starvation and the action of PHLPP1 is required to maintain basal CMA (Arias, 2015). Sustaining a balanced CMA activity is especially relevant for metabolic homeostasis as this degradatory pathway is important for regulating levels of key metabolic enzymes involved in carbohydrate and lipid metabolism (Schneider et al., 2014). Remarkably, treatment of peripheral blood mononuclear cells with an mTORC1/2 inhibitor has been shown to inhibit phosphorylation of Ser473 of AKT and enhance association of AKT with PHLPP1 which may further contribute to the inactivation of AKT (Gupta et al., 2012). Another study suggests that inhibition of mTORC2 via the ATP synthase inhibitor mycotoxin citreoviridin inhibits mTORC2, resulting in apoptosis of cardiomyocytes through lysosomal membrane permeabilization and detrimental autophagy (Feng et al., 2019).

**FIGURE 2:**
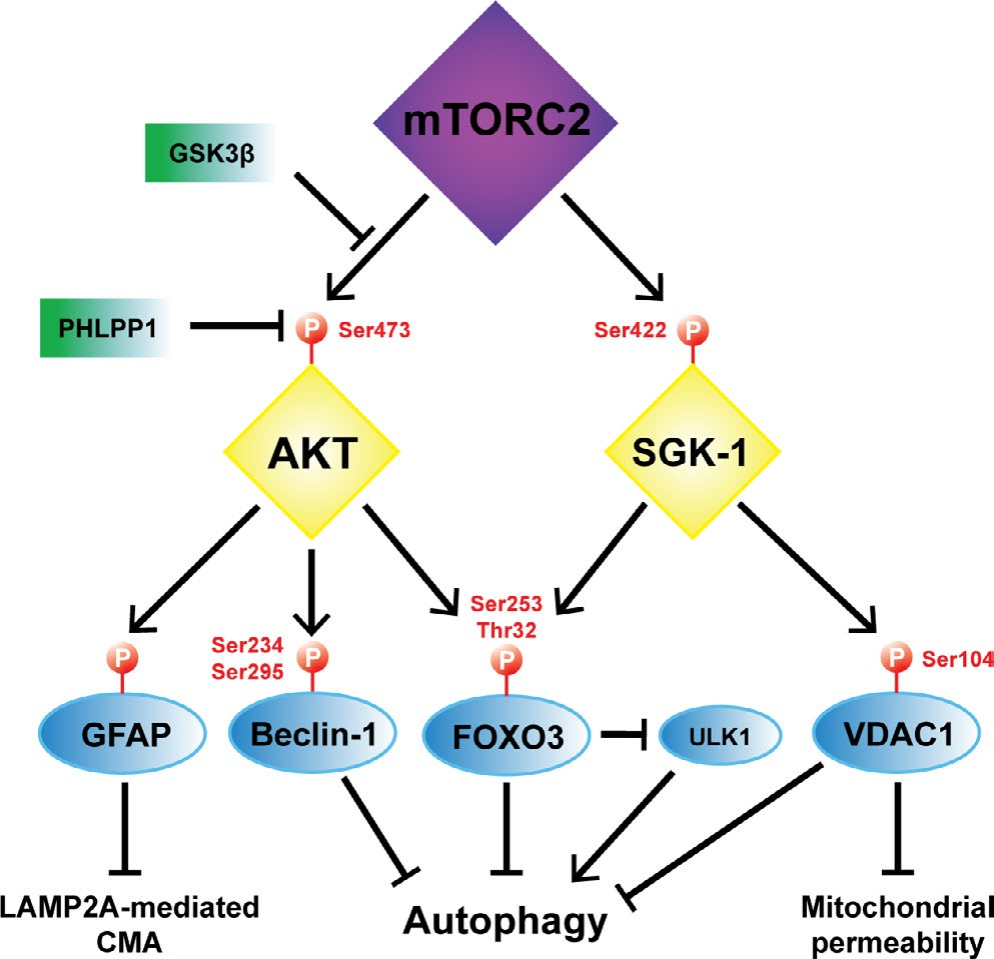
The mTORC2‐AKT‐SGK axis in autophagy. mTORC2 phosphorylates Ser473 of AKT, whereas GSK3b prevents this through an inhibitory phosphorylation of RICTOR. In turn, the phosphatase PHLPP1 can remove the phosphorylation of Ser473 in AKT. pAKT at Ser473 can place inhibitory phosphorylations on Ser234/Ser295 of Beclin‐1 and Ser253/Thr32 of FoxO3, which interferes with the role of these two proteins in the positive modulation of autophagy. pAKT at Ser473 also phosphorylates and inhibits GFAP, resulting in negative regulation of LAMP2A‐mediated CMA. mTORC2 phosphorylates Ser422 of SGK‐1 which, in turn, phosphorylates Thr32 of FoxO3 and Ser104 of VDAC1. pFoxO3 does not participate in the transcriptional upregulation of autophagy and it is believed to reduce the expression of ULK1 and further inhibit autophagy. SGK‐1‐mediated inhibitory phosphorylation of VDAC1 reduces autophagy, mitophagy, and associated mitochondrial permeability

Glycogen synthase kinase 3 beta (GSK3β), a kinase known to participate in several pathways connected with autophagy regulation, can phosphorylate Ser1235 of RICTOR and abrogate binding and phosphorylation of AKT by mTORC2 (Chen et al., 2011). ShRNA‐mediated silencing of RICTOR or its downregulation through the action of microRNAs such as miR211 or miR15a and miR16 have been reported to activate autophagy, presumably through the downstream inactivation of mTORC1 (Huang et al., 2015; Ozturk et al., 2019). Active mTORC2 induces the phosphorylation of AKT at Ser473, a step needed for full activation of AKT (Sarbassov et al., 2005), enhancing its activity in a substrate‐dependent manner. Deletion of RICTOR or mTOR‐associated protein, LST8 homolog (mLST8) ablated insulin‐induced phosphorylation of Forkhead box class O 3 (FOXO3), whereas this did not affect phosphorylation of TSC2 or Ser9 of GSK3β (Guertin et al., 2006). These findings suggest that although Ser473 phosphorylation is required for AKT‐mediated phosphorylation of FOXO3 or PKCα, it is not required for that of TSC2, a well‐described mTORC1 inhibitor (Saxton & Sabatini, 2017; Webb & Brunet, 2014). This is an important observation that suggests that the proposed mTORC2‐AKT‐TSC2 pathway thought to regulate mTORC1 may not be valid (Gaubitz et al., 2016), and that a different pool of phosphorylated AKT is responsible for phosphorylation of TSC2 (Huang & Manning, 2009) (Figure 2).

### 3.2 SIN1‐RB

Sin1 is a protein component specifically found inTORC2 (absent in mTORC1) and is required for the kinase activity of the complex in multiple ways. Sin1 is required for docking the complex to the plasma membrane (Yuan & Guan, 2015). Sin1 knockdown triggers the dissociation of RICTOR from the complex (Yang et al., 2006); however, Sin1 is also responsible for recruiting substrates such as AKT and SGK to mTORC2 for subsequent phosphorylation (Jhanwar‐Uniyal et al., 2017; Liu, Gan et al., 2015; Yang et al., 2006; Yuan & Guan, 2015). Sin1 binds through its PH domain to mTORC2 blocks access to its substrate AKT, whereas PIP_3_ binding to Sin1‐PH releases the tight interaction with mTORC2 and allows the kinase to access substrates such as AKT (Liu, Gan et al., 2015). Accordingly, ablating the particular residues of Sin1 mediating interaction with PIP_3_ inactivates mTORC2 (Liu, Gan et al., 2015). Interestingly, the retinoblastoma (Rb) protein has been found to bind the Sin1‐PH domain, suppressing access of AKT to mTORC2 and its subsequent phosphorylation (Zhang et al., 2016). Conversely, in Rb‐deficient cells, the activity of mTORC2 is enhanced as shown by an increase in the phosphorylation of its downstream targets FOXO and PRAS40 (Zhang et al., 2016). Thus, Rb functions as a positive modulator of autophagy via this interaction and its silencing leads to blockage of autophagic flux in glioblastoma cells, sensitizing them to the action of chemotherapeutic agents (Biasoli et al., 2013).

### 3.3 PKC

One of the first identified substrates of mTORC2 was PKCα, a modulator of actin cytoskeleton reorganization and cell migration (Dos et al., 2004; Hernández‐Negrete et al., 2007; Jacinto et al., 2004,). Whereas deletion of mLST8 or RICTOR, which specifically inactivate mTORC2, compromised the phosphorylation and stability of PKCα (Guertin et al., 2006), mTORC2 has been indicated to be a positive regulator of PKCδ (Gan et al., 2012). The role of different PKC isozymes is under investigation, as contrasting studies indicate that they might be relevant modulators of autophagy (Kaleli et al., 2020). PKCα has been shown to suppress autophagy in breast cancer cells (Wong et al., 2017) and during gestational diabetes, where its action may trigger neural tube defects (Wang et al., 2017). Rapamycin treatment, and resulting inhibition of mTORC2, reduced the phosphorylation and activity of several PKC isozymes, in particular PKCβ, and ameliorated mitochondrial disease and neuropathology in a mouse model of Leigh syndrome (Martin‐Perez et al., 2020). PKCβ pharmacological inhibition or genetic depletion increased autophagy and mitochondrial health (Patergnani et al., 2013). In a contrasting study, PKCα/δ induce phosphorylation of Ser9 and Ser21 in GSK3β residues associated with an inactive form of this kinase (Li et al., 2016). Inactive GSK3β has been shown to contribute to nuclear translocation and activation of TFEB, resulting in autophagy induction (Li et al., 2018; Marchand et al., 2015). An additional mechanism by which PKCδ may be involved in the positive regulation of autophagy is via activation of c‐Jun N‐terminal kinases (JNK) and p38 MAPK which subsequently phosphorylate zinc finger with KRAB And SCAN domains 3 (ZKSCAN3), resulting in cytoplasmic translocation and inactivation of this transcriptional repressor of autophagy (Li et al., 2016). Taken in total, these data suggest that in the context of autophagy regulation, the interaction between the mTORC2 and different PKC isozymes is complex and likely dependent on crosstalk with other connected signaling pathways.

### 3.4 SGK‐1

Serum and glucocorticoid‐induce protein kinase is another member of the AGC subfamily of kinases which has significant sequence homology (~54%) with AKT (Kobayashi & Cohen, 1999; Liu et al., 2017). SGK‐1 is phosphorylated at Ser422 in its hydrophobic region by mTORC2 (Luo et al., 2018) and functions as an effector modulated by the insulin/IGF‐1 pathway, involved in the stress response through the negative regulation of the skinhead 1 (skn‐1)/nuclear factor erythroid 2‐related factor 2 (NRF2) pathway (Aspernig et al., 2019; Heimbucher et al., 2020; Mizunuma et al., 2014).

Due to its high structural homology, SGK‐1 has been shown to display significant overlap with AKT in the subset of target proteins that they both regulate via phosphorylation. Some of these shared downstream effectors include GSK3β (Kobayashi & Cohen, 1999) and FOXO3 (Brunet et al., 2001). As a putative negative regulator of FOXO3, the role of SGK‐1 in autophagy is the subject of current investigation. Pharmacological inhibition of SGK‐1 results in an increase in protein expression of ULK1, a major initiator of the autophagy process. Although the mechanism for the increased expression of ULK1 upon SGK‐1 inhibition remains elusive, it has been hypothesized that it takes place through the inhibitory phosphorylation of FOXO3 by SGK‐1 (Maestro et al., 2020; Zuleger et al., 2018). Interestingly, genetic disruption of the PDK1 interacting fragment (PIF)‐pocket catalytic domain of PDK1, which abrogates activation of SGK‐1, RSK, and S6K but not AKT, do not result in overall reduced phosphorylation of GSK3 or FOXO (Collins et al., 2003), suggesting that SGK‐1 might not be the key driver in their activation, possibly due to functional overlap with other AGC kinases. Defining the specific targets of SGK‐1 activity and their biological effects has been challenging due to the lack of effective and specific inhibitors (Di Cristofano, 2017). Downregulation of sgk‐1 activity through direct knockout/knockdown of sgk‐1 or through ablation of rict‐1 and associated mTORC2 inhibition in *C*. *elegans* induces autophagy, as demonstrated by increased expression of the microtubule‐associated proteins 1A/1B light chain 3 (LC3A/B) ortholog LC3 GABARAP and GATE‐16 family 1 (lgg‐1) and the TFEB ortholog helix loop helix 30 (hlh‐30). The mRNA expression of both *lgg*‐*1* and *hlh*‐*30* is upregulated upon voltage‐dependent anion channel 1 (VDAC1) overexpression, a protein that in turn is phosphorylated by sgk‐1 and subsequently targeted for degradation. Surprisingly, these mutants displayed a shortened lifespan. The authors showed that VDAC1 concomitantly activates mitochondrial permeability to initiate mitophagy, highlighting the potential deleterious effects of elevated levels of autophagy *in vivo* that can disrupt mitochondrial homeostasis (Zhou et al., 2019).

Oxidative stress has been identified as an inducer of mTORC2 activity in myofibroblasts (Bernard et al., 2020). Interestingly, Aspernig et al. showed that the mTORC2‐SGK‐1 axis is involved in maintaining mitochondrial homeostasis, whereas mTORC2 inhibits autophagy through activation of AKT and inactivation of FOXO (Aspernig et al., 2019). From these observations, we propose that a useful strategy to benefit from elevated autophagy flux while counteracting the harmful effects of mTORC2‐SGK‐1 inhibition would be the preservation of specific targets downstream of SGK‐1 that suppress mitochondrial permeability (Figure 2).

### 3.5 The foxos

The FOXO family of transcription factors includes several related proteins in mammals such as FOXO1, 3, 4, and 6 and orthologs across the animal kingdom. They contain a forkhead box DNA‐binding domain and nuclear localization and export signals that direct the nuclear shuttling and cytoplasmic export of these factors (Cheng, 2019). FOXOs have been associated with autophagy regulation in both transcription‐dependent and independent fashions. The factors can bind to the promoters of autophagy‐related genes through their DNA‐binding domain (Liu et al., 2018; Webb & Brunet, 2014). Cytoplasmic FOXO1 interacts with ATG7, a ubiquitin‐activating E1‐like enzyme required for autophagosome assembly (Xiong, 2015; Zhao et al., 2010). FOXO1 has been found to be acetylated by cyclic AMP (cAMP)‐response element‐binding protein (CREB)‐binding protein paralog p300 and phosphorylated by AKT. These modifications may facilitate its cytoplasmic retention and interaction with ATG7 (Shen et al., 2017). On the other hand, deacetylases like SIRT1/2 induce nuclear translocation of FOXO1/3 and transcriptional activation of target genes (Liu, Bi et al., 2015). SIRT1 was found to induce the transcription of RICTOR, phosphorylation of Ser473 of Akt and Ser253 of Foxo1 at S253, which results in decreased gluconeogenesis (Tobita et al., 2016; Wang et al., 2011). In addition to a role for mTORC2 as a regulator of FOXO through AKT‐induced phosphorylation and cytoplasmic localization, mTORC2 has been shown to directly interact with and suppress SIRT6, whereas inactivation through RICTOR knockdown lifts SIRT6 suppression and promotes FOXO1 deacetylation and nuclear translocation (Jung et al., 2019). These findings suggest that a fine‐tuned control of FOXO1 activity through post‐translational modification and subsequent changes in subcellular localization may lead to different roles in autophagy regulation (Cheng, 2019).

FOXO3 has been shown to drive autophagy in hematopoietic stem cells (HSCs) and to be a key player in the regulation of autophagy in muscle tissue. *FOXO3a* ^−/−^ knockout in HSCs leads to significant downregulation of the expression of autophagy‐related genes paralleled with an increase in LC3 protein levels that suggest impaired autophagic flux (Warr et al., 2013).

FOXO3 downstream of the mTORC2‐AKT axis is sufficient to drive autophagy in muscle cells (Mammucari et al., 2007; Zuleger et al., 2018). Two identified target effectors of FOXO3‐driven autophagy are LC3 and B‐cell lymphoma 2 (BCL2)‐interacting protein 3 (BNIP3). Conversely, mTORC2‐AKT phosphorylates and inactivates FOXO3 and autophagy independently of mTORC1 and is not prevented by rapamycin (Mammucari et al., 2007). SGK‐1 can also phosphorylate and inactivate FOXO3. Interestingly, phosphorylated FOXO3a has been reported to interact with LC3. Inhibiting SGK‐1 leads to reduced levels of p‐FOXO3 interacting with LC3 and increased LC3‐I/LC3‐II conversion, suggesting increased autophagic flux (Liu et al., 2017). In a rat model of burn‐induced muscle wasting, down‐regulating Phlpp1 led to elevated Akt and FoxO3 phosphorylation, which in turn suppressed detrimental autophagy and mitigated muscle wasting (Yu et al., 2019b; Zhao et al., 2007). Another mechanism by which mTORC2 regulates FOXO3 activity is through phosphorylation and inactivation of histone deacetylase (HDAC), resulting in increased acetylation of FOXO3 and reduced transcriptional activity (Masui et al., 2013).

**FIGURE 3:**
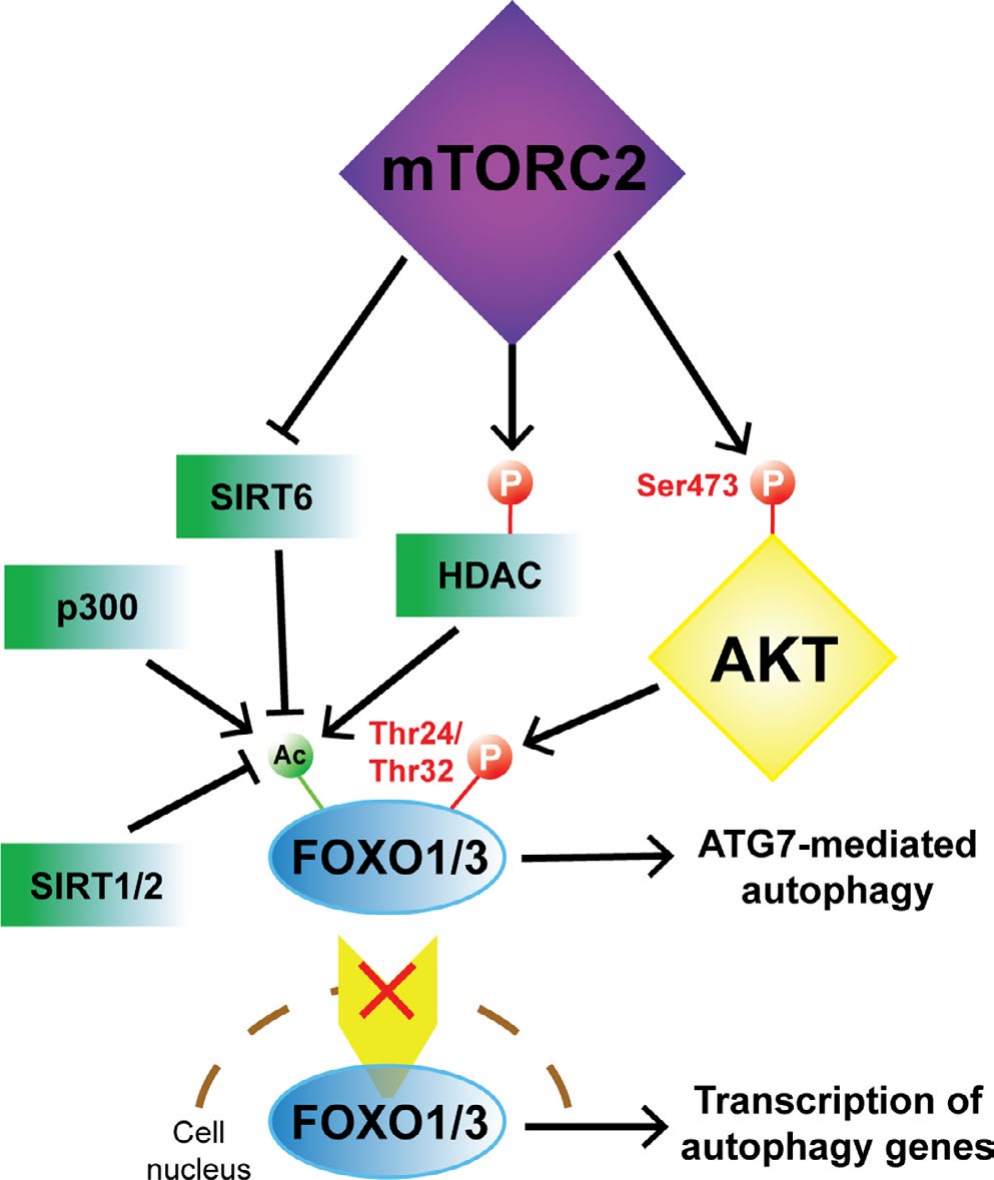
mTORC2‐mediated regulation of FoxO1/3 subcellular localization and activity in autophagy. mTORC2‐mediated phosphorylation of Ser473 in AKT triggers the phosphorylation of Thr24 in FoxO1 and its equivalent Thr32 in FoxO3. mTORC2 phosphorylates and inactivates the deacetylase HDAC contributing to the acetylation of FoxO1/3. Similarly, mTORC2 directly interacts with SIRT6 and inhibits its deacetylase activity on FoxO1/3. Independently of mTORC2, FoxO1/3 is acetylated by p300 and deacetylated by SIRT1/2. Phosphorylation and/or acetylation of FoxO1/3 promote their cytoplasmic retention and interaction with ATG7 which has a role in inducing autophagy. In contrast, dephosphorylated and deacetylated FoxO1/3 are readily transported in the nucleus where they stimulate the transcription of autophagy‐related genes

Another study shed additional light on mechanism involved in autophagy regulation by FOXO3, involving glutamine metabolism as a regulator of mTORC1 localization. FOXO3 can induce the expression of glutamine synthetase, leading to rising glutamine levels that, in turn, can inhibit mTORC1 localization to the lysosomal membrane impairing the ability of mTORC1 to function as an autophagy negative regulator (van der Vos et al., 2012) (Figure 3).

### 3.6 AMPK

In endothelial cells, silencing of RICTOR increased the expression of LC3‐II while it decreased protein levels of p62, two markers that are associated with enhanced autophagic flux, in parallel with AMP‐activated protein kinase (AMPK) activation. In addition, arginase 2 (ARG2) can inactivate autophagy through positive regulation of mTORC2 and subsequent inactivation of AMPK (Xiong et al., 2014). AMPK appears to participate in a regulatory feedback loop as activating AMPK with metformin, a drug employed in the treatment of type 2 diabetes, increased liver mTORC2 *in vivo* (Kazyken et al., 2019). Metformin‐mediated AMPK activation has been shown to induce autophagosome maturation and induce cell‐cycle arrest through combined mTORC1/2 repression (Jang et al., 2018; Wang et al., 2018).

Additionally, AMPK has been identified as an upstream regulator of the MEK/ERK pathway which, in turn, can disassemble and inhibit both mTOR complexes and induce autophagy through upregulation of Beclin‐1. Interestingly, the authors of this study suggested that whereas individually inhibiting mTORC1 or mTORC2 results in moderate activation of Beclin‐1 and protective autophagy, dual inhibition of mTORC1/2 and the subsequent robust activation of Beclin‐1 can result in destructive autophagy (Wang et al., 2009).

## 4 UNVEILING THE ROLE OF mTORC2 IN AUTOPHAGY: TOWARD SPECIFIC mTORC2 INHIBITION

Autophagy has long been targeted as a modulatable cellular process with vast consequences in age‐related diseases including cancer and neurodegeneration and also in lifespan extension. In particular, downregulation of mTOR activity using the specific inhibitor rapamycin and, more recently, newly developed rapalogs has led to promising advances in this quest (Blagosklonny, 2012). Rapamycin is traditionally considered a specific mTORC1 inhibitor, while not impacting on mTORC2 activity (Dos et al., 2004). However, recent studies have shown that whereas acute exposure to the inhibitor mainly leads to inhibition of mTORC1, chronic exposure results in additional inhibition of mTORC2, an important observation for long‐term studies using this molecule including lifespan extension research. Long‐term administration of rapamycin was shown to disrupt mTORC2, induce insulin resistance and thus abrogate lifespan extension in male mice, whereas selective mTORC1 genetic ablation increased it (Lamming et al., 2012). Similarly, depletion of Rictor decreases male lifespan in mice (Lamming et al., 2014). Intermittent dosing of rapamycin has been proposed as a means to prevent unwanted mTORC2 inhibition and the resulting metabolic defects *in vivo* (Arriola Apelo et al., 2016). Interestingly, whereas male mice gonadectomy did not revert lifespan shortening in male mice upon chronic rapamycin administration, ovariectomy promoted lifespan in female mice lacking hepatic Rictor (Apelo et al., 2020). In line with these findings, mice with hypothalamic Rictor deficiency display increased diet‐induced obesity, frailty, and survival (Chellappa et al., 2019). By contrast, other studies suggest that rapamycin administration in late‐life increases the lifespan of males and female mice (Harrison et al., 2009), and that dietary intervention with Torin1, a dual mTORC1/2 inhibitor, resulted in prolonged lifespan in *D*. *melanogaster* with no reduction in fertility (Mason et al., 2018). Intriguingly, the effects of inhibiting mTORC2 in *C*. *elegans* lifespan are contradictory. Whereas loss of sgk‐1 has been shown to extend lifespan (Hertweck et al., 2004), depleting the *C*. *elegans* RICTOR homolog rict‐1 or sgk‐1 reduced lifespan in a diet‐dependent fashion (Soukas et al., 2009). Furthermore, Akt1 haploinsufficiency alone leads to extended lifespan in mice (Nojima et al., 2013).

Torin1, an ATP‐competitive compound that inhibits the catalytic activity of both mTORC1 and mTORC2 complexes, has been shown to induce autophagy in a more robust manner than rapamycin which may suggest that combined inhibition of both complexes is more efficient at inducing the autophagy process (Oh & Jacinto, 2011; Thoreen et al., 2009). However, rapamycin‐insensitive/torin1‐sensitive functions of mTORC1 cannot be ruled out when studying the differential effects of these two inhibitors on autophagy. Upon Torin1 treatment, RICTOR‐deficient engineered MEF cells showed a more robust inhibition of protein translation coupled with activation of autophagy than what was observed upon rapamycin treatment, indicating that the distinctive biological effects of Torin1 as compared to rapamycin might not solely depend on mTORC2 activity (Thoreen & Sabatini, 2009). More recently, efforts directed to the development of inhibitors that differentially target mTORC1 or mTORC2 are underway (Mahoney et al., 2018; Schreiber et al., 2019). A novel small molecule that interferes with the association of RICTOR with mTOR has been identified as a potential specific mTORC2 inhibitor in glioblastoma. Importantly, the compound has shown inhibitory activity on AKT, PKCα, and N‐Myc Downstream Regulated 1 (NDRG1) while mTORC1 remained unaffected (Benavides‐Serrato et al., 2017).

## 5 CONFLICTING VIEWS ON THE ROLE OF mTORC2 IN RELATION TO AUTOPHAGY

The dual mTORC1/2 inhibitor OSI‐027 has been reported to induce extensive macropinocytosis in rhabdomyosarcoma, leading to cell death and halt of tumor growth. Whereas rapamycin treatment did not induce the formation of macropinocytic vacuoles in these cells, RICTOR knockdown recapitulated the effects of the OSI‐027 inhibitor and combined rapamycin treatment and RICTOR knockdown enhanced this effect. These results suggest that inhibition of mTORC2 is responsible for inducing the formation of these vesicles and that inhibition of both mTOR complexes may trigger excessive macropinocytosis with deleterious effects for cellular viability (Srivastava et al., 2019). In the same vein, OSI‐027 has been shown to induce apoptosis and autophagy in a battery of lymphoid tumoral cell lines and clinical samples, in a manner more effective than rapamycin (Gupta et al., 2012). However, in this study, autophagy is suspected to play a protective role that counteracts excessive apoptosis induction, as combined treatment with chloroquine further increased cell death. Another interesting observation in this study was that whereas OSI‐027 treatment inhibited several mTORC1/2 targets including S6K1, 4EBP1, phosphorylated Ser473 of AKT and FOXO3a; TSC2, GSK3β, and FOXO1 remained unaffected (Gupta et al., 2012), indicating that the regulation of its target genes by mTORC2 is complex and may be dependent on its interaction with interconnected pathways and the cellular or environmental context.

While multiple studies indicate a role for mTOR as a negative modulator of autophagy not only assembled as complex 1 but also as part of complex 2, opposing views on the role of mTORC2 in the regulation of autophagy have been presented. Knockdown of the *daw* gene in *D*. *melanogaster* has been proposed to enhance autophagy and cardiac health through increasing the expression of Rictor, while depleting Rictor blunted this effect (Chang et al., 2020). A temperature‐sensitive knockdown of mTORC2 or depletion of the yeast SGK‐1 ortholog ypk1 greatly reduced the autophagic response upon amino acid starvation in *S*. *cerevisiae*. The induction of autophagy through mTORC2‐ypk1 was shown to be dependent on the role of calcineurin as a negative autophagy regulator (Vlahakis et al., 2014). In contrast, calcineurin is a phosphatase that has been described as a positive regulator of autophagy by dephosphorylating and promoting nuclear localization of TFEB in mammals (Medina et al., 2015), suggesting that the conflicting views on the role of the phosphatase or the mTORC2‐ypk1 axis might be largely organism‐dependent.

## 6 CONCLUSIONS

The abundant biological effects of the mTOR kinase as part of both mTORC1/2 complexes are far from being fully elucidated and highlight the convergence of an intricate network of signaling and metabolic pathways. Several key molecular players that regulate autophagy‐related pathways, a biological process of particular interest regarding its potential as a therapeutic target, have been shown to be directly involved with the activity of mTORC2. Broad genetic or pharmacological inactivation of mTORC2 could carry complex biological effects that go beyond its activity as a mere kinase, whereas fine‐tuning its ability for recruiting particular substrates through development of novel specific inhibitors or genetic interventions would prove useful to pinpoint the mechanistic effects of the individual components within the complex and the action of the multiple downstream effectors which are, in addition, dependent on the cellular context and the nutritional status. Dissecting the various inputs and outputs of mTORC2 will be crucial to understanding its role in the regulation of autophagy and the role of autophagy itself in human health and disease.

## CONFLICT OF INTEREST

The authors declare no competing financial interests.

## AUTHOR CONTRIBUTIONS

J.B. conceived the manuscript, prepared figures, and wrote the manuscript. J.K.A. wrote and revised the manuscript.

## References

[acel13431-bib-0001] Amaravadi, R. K., Lippincott‐Schwartz, J., Yin, X.‐M., Weiss, W. A., Takebe, N., Timmer, W., DiPaola, R. S., Lotze, M. T., & White, E. (2011). Principles and current strategies for targeting autophagy for cancer treatment. Clinical Cancer Research, 17(4), 654–666. 10.1158/1078-0432.CCR-10-2634 21325294 PMC3075808

[acel13431-bib-0002] Arriola Apelo, S. I., Lin, A., Brinkman, J. A., Meyer, E., Morrison, M., Tomasiewicz, J. L., Pumper, C. P., Baar, E. L., Richardson, N. E., Alotaibi, M., & Lamming, D. W. (2020). Ovariectomy uncouples lifespan from metabolic health and reveals a sex‐hormone‐dependent role of hepatic mTORC2 in aging. eLife, 9, e56177. 10.7554/elife.56177 32720643 PMC7386906

[acel13431-bib-0003] Arias, E. (2015). Lysosomal mTORC2/PHLPP1/Akt axis: a new point of control of chaperone‐mediated autophagy. Oncotarget, 6(34), 35147–35148. 10.18632/oncotarget.5903 26436591 PMC4742091

[acel13431-bib-0004] Arias, E., Koga, H., Diaz, A., Mocholi, E., Patel, B., & Cuervo, A. M. (2015). Lysosomal mTORC2/PHLPP1/Akt Regulate Chaperone‐Mediated Autophagy. Molecular Cell, 59(2), 270–284. 10.1016/j.molcel.2015.05.030 26118642 PMC4506737

[acel13431-bib-0005] Arriola Apelo, S. I., Neuman, J. C., Baar, E. L., Syed, F. A., Cummings, N. E., Brar, H. K., Pumper, C. P., Kimple, M. E., & Lamming, D. W. (2016). Alternative rapamycin treatment regimens mitigate the impact of rapamycin on glucose homeostasis and the immune system. Aging Cell, 15(1), 28–38. 10.1111/acel.12405 26463117 PMC4717280

[acel13431-bib-0006] Aspernig, H., Heimbucher, T., Qi, W., Gangurde, D., Curic, S., Yan, Y., Donner von Gromoff, E., Baumeister, R., & Thien, A. (2019). Mitochondrial Perturbations Couple mTORC2 to Autophagy in *C. elegans* . Cell Reports, 29(6), 1399–1409. 10.1016/j.celrep.2019.09.072 31693882

[acel13431-bib-0007] Bartolome, A., & Guillen, C. (2014). Role of the mammalian target of rapamycin (mTOR) complexes in pancreatic beta‐cell mass regulation. Vitamins and Hormones, 2014(95), 425–469. 10.1016/B978-0-12-800174-5.00017-X 24559928

[acel13431-bib-0008] Benavides‐Serrato, A., Lee, J., Holmes, B., Landon, K. A., Bashir, T., Jung, M. E., Lichtenstein, A., & Gera, J. (2017). Specific blockade of Rictor‐mTOR association inhibits mTORC2 activity and is cytotoxic in glioblastoma. PLoS One, 12(4), e0176599. 10.1371/journal.pone.0176599 28453552 PMC5409528

[acel13431-bib-0009] Bentley, N. J., Eisen, T., & Goding, C. R. (1994). Melanocyte‐specific expression of the human tyrosinase promoter: activation by the microphthalmia gene product and role of the initiator. Molecular and Cellular Biology, 14(12), 7996–8006. 10.1128/mcb.14.12.7996-8006.1994 7969139 PMC359338

[acel13431-bib-0010] Bento, C. F., Renna, M., Ghislat, G., Puri, C., Ashkenazi, A., Vicinanza, M., Menzies, F. M., & Rubinsztein, D. C. (2016). Mammalian Autophagy: How Does It Work? Annual Review of Biochemistry, 85(1), 685–713. 10.1146/annurev-biochem-060815-014556 26865532

[acel13431-bib-0011] Berchtold, D., Piccolis, M., Chiaruttini, N., Riezman, I., Riezman, H., Roux, A., Walther, T. C., & Loewith, R. (2012). Plasma membrane stress induces relocalization of Slm proteins and activation of TORC2 to promote sphingolipid synthesis. Nature Cell Biology, 14(5), 542–547. 10.1038/ncb2480 22504275

[acel13431-bib-0012] Bernard, M., Dieudé, M., Yang, B., Hamelin, K., Underwood, K., & Hébert, M. J. (2014). Autophagy fosters myofibroblast differentiation through MTORC2 activation and downstream upregulation of CTGF. Autophagy, 10(12), 2193–2207. 10.4161/15548627.2014.981786 25495560 PMC4502773

[acel13431-bib-0013] Bernard, M., Yang, B., Migneault, F., Turgeon, J., Dieudé, M., Olivier, M.‐A., Cardin, G. B., El‐Diwany, M., Underwood, K., Rodier, F., & Hébert, M.‐J. (2020). Autophagy drives fibroblast senescence through MTORC2 regulation. Autophagy, 16(11), 2004–2016. 10.1080/15548627.2020.1713640 31931659 PMC7595590

[acel13431-bib-0014] Biasoli, D., Kahn, S. A., Cornélio, T. A., Furtado, M., Campanati, L., Chneiweiss, H., Moura‐Neto, V., & Borges, H. L. (2013). Retinoblastoma protein regulates the crosstalk between autophagy and apoptosis, and favors glioblastoma resistance to etoposide. Cell Death & Disease, 4(8), e767. 10.1038/cddis.2013.283 23949216 PMC3763445

[acel13431-bib-0015] Blagosklonny, M. V. (2012). Rapalogs in cancer prevention: Anti‐aging or anticancer? Cancer Biology and Therapy, 13(14), 1349–1354. 23151465 10.4161/cbt.22859 PMC3542224

[acel13431-bib-0016] Brown, E. J., Albers, M. W., Bum Shin, T., ichikawa, K., Keith, C. T., Lane, W. S., & Schreiber, S. L. (1994). A mammalian protein targeted by G1‐arresting rapamycin–receptor complex. Nature, 369(6483), 756–758. 10.1038/369756a0 8008069

[acel13431-bib-0017] Brunet, A., Park, J., Tran, H., Hu, L. S., Hemmings, B. A., & Greenberg, M. E. (2001). Protein Kinase SGK Mediates Survival Signals by Phosphorylating the Forkhead Transcription Factor FKHRL1 (FOXO3a). Molecular and Cellular Biology, 21(3), 952–965. 10.1128/MCB.21.3.952-965.2001 11154281 PMC86685

[acel13431-bib-0018] Burke, P., Schooler, K., & Wiley, H. S. (2001). Regulation of epidermal growth factor receptor signaling by endocytosis and intracellular trafficking. Molecular Biology of the Cell, 12(6), 1897–1910. 11408594 10.1091/mbc.12.6.1897 PMC37350

[acel13431-bib-0019] Cameron, A. J. M., Linch, M. D., Saurin, A. T., Escribano, C., & Parker, P. J. (2011). mTORC2 targets AGC kinases through Sin1‐dependent recruitment. The Biochemical Journal, 439(2), 287–297. 21806543 10.1042/BJ20110678

[acel13431-bib-0020] Carriere, A., Romeo, Y., Acosta‐Jaquez, H. A., Moreau, J., Bonneil, E., Thibault, P., Fingar, D. C., & Roux, P. P. (2011). ERK1/2 Phosphorylate Raptor to Promote Ras‐dependent Activation of mTOR Complex 1 (mTORC1). Journal of Biological Chemistry, 286(1), 567–577. 10.1074/jbc.m110.159046 PMC3013016 21071439

[acel13431-bib-0021] Caviston, J. P., Zajac, A. L., Tokito, M., & Holzbaur, E. L. (2011). Huntingtin coordinates the dynein‐mediated dynamic positioning of endosomes and lysosomes. Molecular Biology of the Cell, 22(4):478–492. 21169558 10.1091/mbc.E10-03-0233 PMC3038646

[acel13431-bib-0022] Chang, K., Kang, P., Liu, Y., Huang, K., Miao, T., Sagona, A. P., Nezis, I. P., Bodmer, R., Ocorr, K., & Bai, H. (2020). TGFB‐INHB/activin signaling regulates age‐dependent autophagy and cardiac health through inhibition of MTORC2. Autophagy, 16(10), 1807–1822. 10.1080/15548627.2019.1704117 31884871 PMC8386626

[acel13431-bib-0023] Chellappa, K., Brinkman, J. A., Mukherjee, S., Morrison, M., Alotaibi, M. I., Carbajal, K. A., Alhadeff, A. L., Perron, I. J., Yao, R., Purdy, C. S., DeFelice, D. M., Wakai, M. H., Tomasiewicz, J., Lin, A., Meyer, E., Peng, Y., Arriola Apelo, S. I., Puglielli, L., Betley, J. N., … Lamming, D. W. (2019). Hypothalamic mTORC2 is essential for metabolic health and longevity. Aging Cell, 18(5), e13014. 10.1111/acel.13014 31373126 PMC6718533

[acel13431-bib-0024] Chen, C.‐H., Shaikenov, T., Peterson, T. R., Aimbetov, R., Bissenbaev, A. K., Lee, S.‐W., Wu, J., Lin, H.‐K., & Sarbassov, D. D. (2011). ER Stress Inhibits mTORC2 and Akt Signaling Through GSK‐3 ‐Mediated Phosphorylation of Rictor. Science Signaling, 4(161), ra10–ra10. 10.1126/scisignal.2001731 21343617

[acel13431-bib-0025] Cheng, Z. (2019). The FoxO–Autophagy Axis in Health and Disease. Trends in Endocrinology and Metabolism, 30(9), 658–671. 10.1016/j.tem.2019.07.009 31443842

[acel13431-bib-0026] Collins, B. J., Deak, M., Arthur, J. S. C., Armit, L. J., & Alessi, D. R. (2003). In vivo role of the PIF‐binding docking site of PDK1 defined by knock‐in mutation. EMBO Journal, 22(16), 4202–4211. 10.1093/emboj/cdg407 PMC175797 12912918

[acel13431-bib-0027] Daher, B., Parks, S. K., Durivault, J., Cormerais, Y., Baidarjad, H., Tambutte, E., Pouysségur, J., & Vučetić, M. (2019). Genetic Ablation of the Cystine Transporter xCT in PDAC Cells Inhibits mTORC1, Growth, Survival, and Tumor Formation via Nutrient and Oxidative Stresses. Cancer Research, 79(15), 3877–3890. 10.1158/0008-5472.CAN-18-3855 31175120

[acel13431-bib-0028] Dai, H., & Thomson, A. W. (2019). The “other” mTOR complex: New insights into mTORC2 immunobiology and their implications. American Journal of Transplantation, 19(6), 1614–1621. 30801921 10.1111/ajt.15320 PMC6538441

[acel13431-bib-0120] Dalle Pezze, P., Ruf, S., Sonntag, A. G., Langelaar‐Makkinje, M., Hall, P., Heberle, A. M., Razquin Navas, P., van Eunen, K., Tölle, R. C., Schwarz, J. J., Wiese, H., Warscheid, B., Deitersen, J., Stork, B., Fäßler, E., Schäuble, S., Hahn, U., Horvatovich, P., Shanley, D. P., & Thedieck, K. (2016). A systems study reveals concurrent activation of AMPK and mTOR by amino acids. Nature Communications, 7, 13254. 10.1038/ncomms13254 PMC5121333 27869123

[acel13431-bib-0029] Dan, H. C., Antonia, R. J., & Baldwin, A. S. (2016). PI3K/Akt promotes feedforward mTORC2 activation through IKKα. Oncotarget, 7(16), 21064–21075. 10.18632/oncotarget.8383 27027448 PMC5008269

[acel13431-bib-0030] Di Cristofano, A. (2017). SGK1: The Dark Side of PI3K Signaling. Current Topics in Developmental Biology, 123, 49–71. 28236975 10.1016/bs.ctdb.2016.11.006 PMC5658788

[acel13431-bib-0031] Dibble, C. C., & Manning, B. D. (2013). Signal integration by mTORC1 coordinates nutrient input with biosynthetic output. Nature Cell Biology, 15(6), 555–564. 23728461 10.1038/ncb2763 PMC3743096

[acel13431-bib-0032] Dos D. Sarbassov, Ali, S. M., Kim, D.‐H., Guertin, D. A., Latek, R. R., Erdjument‐Bromage, H., Tempst, P., & Sabatini, D. M. (2004). Rictor, a Novel Binding Partner of mTOR, Defines a Rapamycin‐Insensitive and Raptor‐Independent Pathway that Regulates the Cytoskeleton. Current Biology, 14(14), 1296–1302. 10.1016/j.cub.2004.06.054 15268862

[acel13431-bib-0033] Dozynkiewicz, M., Jamieson, N., MacPherson, I., Grindlay, J., van den Berghe, P. E., von Thun, A., Morton, J., Gourley, C., Timpson, P., Nixon, C., McKay, C., Carter, R., Strachan, D., Anderson, K., Sansom, O., Caswell, P., & Norman, J. (2012). Rab25 and CLIC3 collaborate to promote integrin recycling from late endosomes/lysosomes and drive cancer progression. Developmental Cell, 22(1), 131–145. 10.1016/j.devcel.2011.11.008 22197222 PMC3507630

[acel13431-bib-0034] Dyachok, J., Earnest, S., Iturraran, E. N., Cobb, M. H., & Ross, E. M. (2016). Amino Acids Regulate mTORC1 by an Obligate Two‐step Mechanism. Journal of Biological Chemistry, 291(43), 22414–22426. 10.1074/jbc.M116.732511 PMC5077182 27587390

[acel13431-bib-0035] Ebner, M., Sinkovics, B., Szczygieł, M., Ribeiro, D. W., & Yudushkin, I. (2017). Localization of mTORC2 activity inside cells. Journal of Cell Biology, 216(2), 343–353. 10.1083/jcb.201610060 PMC5294791 28143890

[acel13431-bib-0036] Erie, C., Sacino, M., Houle, L., Lu, M. L., & Wei, J. (2015). Altered lysosomal positioning affects lysosomal functions in a cellular model of Huntington’s disease. European Journal of Neuroscience, 42(3), 1941–1951. 10.1111/ejn.12957 PMC4523460 25997742

[acel13431-bib-0037] Feng, C., Li, D., Chen, M., Jiang, L., Liu, X., Li, Q., Geng, C., Sun, X., Yang, G., Zhang, L., & Yao, X. (2019). Citreoviridin induces myocardial apoptosis through PPAR‐γ‐mTORC2‐mediated autophagic pathway and the protective effect of thiamine and selenium. Chemico‐Biological Interactions, 311, 108795. 10.1016/j.cbi.2019.108795 31419397

[acel13431-bib-0038] Gan, X., Wang, J., Wang, C., Sommer, E., Kozasa, T., Srinivasula, S., Alessi, D., Offermanns, S., Simon, M. I., & Wu, D. (2012). PRR5L degradation promotes mTORC2‐mediated PKC‐δ phosphorylation and cell migration downstream of Gα12. Nature Cell Biology, 14(7), 686–696. 10.1038/ncb2507 22609986 PMC3389271

[acel13431-bib-0039] Gaubitz, C., Oliveira, T. M., Prouteau, M., Leitner, A., Karuppasamy, M., Konstantinidou, G., Rispal, D., Eltschinger, S., Robinson, G. C., Thore, S., Aebersold, R., Schaffitzel, C., & Loewith, R. (2015). Molecular Basis of the Rapamycin Insensitivity of Target Of Rapamycin Complex 2. Molecular Cell, 58(6), 977–988. 10.1016/j.molcel.2015.04.031 26028537

[acel13431-bib-0040] Gaubitz, C., Prouteau, M., Kusmider, B., & Loewith, R. (2016). TORC2 Structure and Function. Trends in Biochemical Sciences, 41(6), 532–545. 27161823 10.1016/j.tibs.2016.04.001

[acel13431-bib-0041] Gu, Y., Albuquerque, C. P., Braas, D., Zhang, W., Villa, G. R., Bi, J., Ikegami, S., Masui, K., Gini, B., Yang, H., Gahman, T. C., Shiau, A. K., Cloughesy, T. F., Christofk, H. R., Zhou, H., Guan, K.‐L., & Mischel, P. S. (2017). mTORC2 Regulates Amino Acid Metabolism in Cancer by Phosphorylation of the Cystine‐Glutamate Antiporter xCT. Molecular Cell, 67(1), 128–138.e7. 10.1016/j.molcel.2017.05.030 28648777 PMC5521991

[acel13431-bib-0042] Guertin, D. A., Stevens, D. M., Thoreen, C. C., Burds, A. A., Kalaany, N. Y., Moffat, J., Brown, M., Fitzgerald, K. J., & Sabatini, D. M. (2006). Ablation in Mice of the mTORC Components raptor, rictor, or mLST8 Reveals that mTORC2 Is Required for Signaling to Akt‐FOXO and PKCα, but Not S6K1. Developmental Cell, 11(6), 859–871. 10.1016/j.devcel.2006.10.007 17141160

[acel13431-bib-0043] Gupta, M., Hendrickson, A. E. W., Yun, S. S., Han, J. J., Schneider, P. A., Koh, B. D., Stenson, M. J., Wellik, L. E., Shing, J. C., Peterson, K. L., Flatten, K. S., Hess, A. D., Smith, B. D., Karp, J. E., Barr, S., Witzig, T. E., & Kaufmann, S. H. (2012). Dual mTORC1/mTORC2 inhibition diminishes Akt activation and induces Puma‐dependent apoptosis in lymphoid malignancies. Blood, 119(2), 476–487. 10.1182/blood-2011-04-346601 22080480 PMC3257013

[acel13431-bib-0044] Gwinn, D. M., Shackelford, D. B., Egan, D. F., Mihaylova, M. M., Mery, A., Vasquez, D. S., Turk, B. E., & Shaw, R. J. (2008). AMPK Phosphorylation of Raptor Mediates a Metabolic Checkpoint. Molecular Cell, 30(2), 214–226. 10.1016/j.molcel.2008.03.003 18439900 PMC2674027

[acel13431-bib-0045] Hah, Y.‐S., Cho, H. Y., Lim, T.‐Y., Park, D. H., Kim, H. M., Yoon, J., Kim, J. G., Kim, C. Y., & Yoon, T.‐J. (2012). Induction of Melanogenesis by Rapamycin in Human MNT‐1 Melanoma Cells. Annals of Dermatology, 24(2), 151. 10.5021/ad.2012.24.2.151 22577264 PMC3346904

[acel13431-bib-0046] Han, R., Bansal, D., Miyake, K., Muniz, V. P., Weiss, R. M., McNeil, P. L., & Campbell, K. P. (2007). Dysferlin‐mediated membrane repair protects the heart from stress‐induced left ventricular injury. Journal of Clinical Investigation, 117(7), 1805–1813. 10.1172/jci30848 PMC1904311 17607357

[acel13431-bib-0047] Hara, K., Maruki, Y., Long, X., Yoshino, K.‐I., Oshiro, N., Hidayat, S., Tokunaga, C., Avruch, J., & Yonezawa, K. (2002). Raptor, a Binding Partner of Target of Rapamycin (TOR), Mediates TOR Action. Cell, 110(2), 177–189. 10.1016/S0092-8674(02)00833-4 12150926

[acel13431-bib-0048] Hara, K., Yonezawa, K., Weng, Q. P., Kozlowski, M. T., Belham, C., & Avruch, J. (1998). Amino acid sufficiency and mTOR regulate p70 S6 kinase and eIF‐4E BP1 through a common effector mechanism. Journal of Biological Chemistry, 273(23), 14484–14494. 10.1074/jbc.273.23.14484 9603962

[acel13431-bib-0049] Harrison, D. E., Strong, R., Sharp, Z. D., Nelson, J. F., Astle, C. M., Flurkey, K., Nadon, N. L., Wilkinson, J. E., Frenkel, K., Carter, C. S., Pahor, M., Javors, M. A., Fernandez, E., & Miller, R. A. (2009). Rapamycin fed late in life extends lifespan in genetically heterogeneous mice. Nature, 460(7253), 392–395. 10.1038/nature08221 19587680 PMC2786175

[acel13431-bib-0050] Hay, N., & Sonenberg, N. (2004). Upstream and downstream of mTOR. Genes & Development, 18(16), 1926–1945. 15314020 10.1101/gad.1212704

[acel13431-bib-0051] Heimbucher, T., Qi, W., & Baumeister, R. (2020). TORC2‐SGK‐1 signaling integrates external signals to regulate autophagic turnover of mitochondria via mtROS. Autophagy, 16(6), 1154–1156. 32293958 10.1080/15548627.2020.1749368 PMC7469665

[acel13431-bib-0052] Heitman, J., Movva, N. R., & Hall, M. N. (1991). Targets for cell cycle arrest by the immunosuppressant rapamycin in yeast. Science, 253(5022), 905–909. 1715094 10.1126/science.1715094

[acel13431-bib-0053] Hernández‐Negrete, I., Carretero‐Ortega, J., Rosenfeldt, H., Hernández‐Garciía, R., Calderón‐Salinas, J. V., Reyes‐Cruz, G., Gutkind, J. S., & Vázquez‐Prado, J. (2007). P‐Rex1 Links Mammalian Target of Rapamycin Signaling to Rac Activation and Cell Migration. Journal of Biological Chemistry, 282(32), 23708–23715. 10.1074/jbc.M703771200 17565979

[acel13431-bib-0054] Hertweck, M., Göbel, C., & Baumeister, R. (2004). C. elegans SGK‐1 is the critical component in the Akt/PKB kinase complex to control stress response and life span. Developmental Cell, 6(4), 577–588. 10.1016/S1534-5807(04)00095-4 15068796

[acel13431-bib-0055] Hosokawa, N., Hara, T., Kaizuka, T., Kishi, C., Takamura, A., Miura, Y., Iemura, S.‐I., Natsume, T., Takehana, K., Yamada, N., Guan, J.‐L., Oshiro, N., & Mizushima, N. (2009). Nutrient‐dependent mTORC1 Association with the ULK1–Atg13–FIP200 Complex Required for Autophagy. Molecular Biology of the Cell, 20(7), 1981–1991. 10.1091/mbc.e08-12-1248 19211835 PMC2663915

[acel13431-bib-0056] Huang, J., & Manning, B. D. (2009). A complex interplay between Akt, TSC2 and the two mTOR complexes. Biochemical Society Transactions, 37(Pt 1), 217–222. 19143635 10.1042/BST0370217 PMC2778026

[acel13431-bib-0057] Huang, N., Wu, J., Qiu, W., Lyu, Q., He, J., Xie, W., Xu, N., & Zhang, Y. (2015). MiR‐15a and miR‐16 induce autophagy and enhance chemosensitivity of Camptothecin. Cancer Biology & Therapy, 16(6), 941–948. 10.1080/15384047.2015.1040963 25945419 PMC4622988

[acel13431-bib-0058] Ikenoue, T., Inoki, K., Yang, Q., Zhou, X., & Guan, K. L. (2008). Essential function of TORC2 in PKC and Akt turn motif phosphorylation, maturation and signalling. EMBO Journal, 27(14), 1919–1931. 10.1038/emboj.2008.119 PMC2486275 18566587

[acel13431-bib-0059] Inoki, K., & Guan, K. L. (2009). Tuberous sclerosis complex, implication from a rare genetic disease to common cancer treatment. Human Molecular Genetics, 18(R1), R94–R100. 10.1093/hmg/ddp032 19297407 PMC2657945

[acel13431-bib-0060] Jacinto, E., Loewith, R., Schmidt, A., Lin, S., Rüegg, M. A., Hall, A., & Hall, M. N. (2004). Mammalian TOR complex 2 controls the actin cytoskeleton and is rapamycin insensitive. Nature Cell Biology, 6(11), 1122–1128. 10.1038/ncb1183 15467718

[acel13431-bib-0061] Jang, M., Park, R., Kim, H., Namkoong, S., Jo, D., Huh, Y. H., Jang, I.‐S., Lee, J. I., & Park, J. (2018). AMPK contributes to autophagosome maturation and lysosomal fusion. Scientific Reports, 8(1). 10.1038/s41598-018-30977-7 PMC6107659 30140075

[acel13431-bib-0062] Jhanwar‐Uniyal, M., Amin, A. G., Cooper, J. B., Das, K., Schmidt, M. H., & Murali, R. (2017). Discrete signaling mechanisms of mTORC1 and mTORC2: Connected yet apart in cellular and molecular aspects. Advances in Biological Regulation, 64, 39–48. 28189457 10.1016/j.jbior.2016.12.001

[acel13431-bib-0063] Jia, R., & Bonifacino, J. S. (2019). Lysosome Positioning Influences mTORC2 and AKT Signaling. Molecular Cell, 75(1), 26–38. 10.1016/j.molcel.2019.05.009 31130364 PMC7446307

[acel13431-bib-0064] Jin, S., DiPaola, R. S., Mathew, R., & White, E. (2007). Metabolic catastrophe as a means to cancer cell death. Journal of Cell Science, 120(Pt 3), 379–383. 10.1242/jcs.03349 17251378 PMC2857576

[acel13431-bib-0065] Jung, S. M., Hung, C.‐M., Hildebrand, S. R., Sanchez‐Gurmaches, J., Martinez‐Pastor, B., Gengatharan, J. M., Wallace, M., Mukhopadhyay, D., Martinez Calejman, C., Luciano, A. K., Hsiao, W.‐Y., Tang, Y., Li, H., Daniels, D. L., Mostoslavsky, R., Metallo, C. M., & Guertin, D. A. (2019). Non‐canonical mTORC2 Signaling Regulates Brown Adipocyte Lipid Catabolism through SIRT6‐FoxO1. Molecular Cell, 75(4), 807–822. 10.1016/j.molcel.2019.07.023 31442424 PMC7388077

[acel13431-bib-0066] Kaleli, H. N., Ozer, E., Kaya, V. O., & Kutlu, O. (2020). Protein Kinase C Isozymes and Autophagy during Neurodegenerative Disease Progression. Cells, 9(3), 553. 10.3390/cells9030553 PMC7140419 32120776

[acel13431-bib-0067] Kaushik, S., & Cuervo, A. M. (2012). Chaperone‐mediated autophagy: A unique way to enter the lysosome world. Trends in Cell Biology, 22(8), 407–417. 22748206 10.1016/j.tcb.2012.05.006 PMC3408550

[acel13431-bib-0068] Kazyken, D., Magnuson, B., Bodur, C., Acosta‐Jaquez, H. A., Zhang, D., Tong, X., Barnes, T. M., Steinl, G. K., Patterson, N. E., Altheim, C. H., Sharma, N., Inoki, K., Cartee, G. D., Bridges, D., Yin, L., Riddle, S. M., & Fingar, D. C. (2019). AMPK directly activates mTORC2 to promote cell survival during acute energetic stress. Science Signaling, 12(585), eaav3249. 10.1126/scisignal.aav3249 31186373 PMC6935248

[acel13431-bib-0069] Knudsen, J. R., Fritzen, A. M., James, D. E., Jensen, T. E., Kleinert, M., & Richter, E. A. (2020). Growth Factor‐Dependent and ‐Independent Activation of mTORC2. Trends in Endocrinology & Metabolism, 31(1), 13–24. 10.1016/j.tem.2019.09.005 31699566

[acel13431-bib-0070] Kobayashi, T., & Cohen, P. (1999). Activation of serum‐ and glucocorticoid‐regulated protein kinase by agonists that activate phosphatidylinositide 3‐kinase is mediated by 3‐phosphoinositide‐dependent protein kinase‐1 (PDK1) and PDK2. Biochemical Journal, 339(2), 319. 10.1042/0264-6021:3390319 PMC1220160 10191262

[acel13431-bib-0071] Komatsu, M., Waguri, S., Koike, M., Sou, Y.‐S., Ueno, T., Hara, T., Mizushima, N., Iwata, J.‐I., Ezaki, J., Murata, S., Hamazaki, J., Nishito, Y., Iemura, S.‐I., Natsume, T., Yanagawa, T., Uwayama, J., Warabi, E., Yoshida, H., Ishii, T., … Tanaka, K. (2007). Homeostatic levels of p62 control cytoplasmic inclusion body formation in autophagy‐deficient mice. Cell, 131(6), 1149–1163. 10.1016/j.cell.2007.10.035 18083104

[acel13431-bib-0072] Kumar, A., Harris, T. E., Keller, S. R., Choi, K. M., Magnuson, M. A., & Lawrence, J. C. (2008). Muscle‐specific deletion of rictor impairs insulin‐stimulated glucose transport and enhances basal glycogen synthase activity. Molecular and Cellular Biology, 28(1), 61–70. 10.1128/MCB.01405-07 17967879 PMC2223287

[acel13431-bib-0073] Lamming, D. W., Mihaylova, M. M., Katajisto, P., Baar, E. L., Yilmaz, O. H., Hutchins, A., Gultekin, Y., Gaither, R., & Sabatini, D. M. (2014). Depletion of Rictor, an essential protein component of m TORC 2, decreases male lifespan. Aging Cell, 13(5), 911–917. 10.1111/acel.12256 25059582 PMC4172536

[acel13431-bib-0074] Lamming, D. W., Ye, L., Katajisto, P., Goncalves, M. D., Saitoh, M., Stevens, D. M., Davis, J. G., Salmon, A. B., Richardson, A., Ahima, R. S., Guertin, D. A., Sabatini, D. M., & Baur, J. A. (2012). Rapamycin‐Induced Insulin Resistance Is Mediated by mTORC2 Loss and Uncoupled from Longevity. Science, 335(6076), 1638–1643. 10.1126/science.1215135 22461615 PMC3324089

[acel13431-bib-0075] Lampada, A., Hochhauser, D., & Salomoni, P. (2017). Autophagy and receptor tyrosine kinase signalling: A mTORC2 matter. Cell Cycle, 16(20), 1855–1856. 28934018 10.1080/15384101.2017.1372548 PMC5638352

[acel13431-bib-0076] Lampada, A., O’Prey, J., Szabadkai, G., Ryan, K. M., Hochhauser, D., & Salomoni, P. (2017). MTORC1‐independent autophagy regulates receptor tyrosine kinase phosphorylation in colorectal cancer cells via an mTORC2‐mediated mechanism. Cell Death and Differentiation, 24(6), 1045–1062. 28475179 10.1038/cdd.2017.41 PMC5442471

[acel13431-bib-0077] Lee, S., Sato, Y., & Nixon, R. A. (2011). Lysosomal proteolysis inhibition selectively disrupts axonal transport of degradative organelles and causes an Alzheimer’s‐like axonal dystrophy. Journal of Neuroscience, 31(21), 7817–7830. 10.1523/JNEUROSCI.6412-10.2011 21613495 PMC3351137

[acel13431-bib-0078] Li, L., Friedrichsen, H. J., Andrews, S., Picaud, S., Volpon, L., Ngeow, K., Berridge, G., Fischer, R., Borden, K. L. B., Filippakopoulos, P., & Goding, C. R. (2018). A TFEB nuclear export signal integrates amino acid supply and glucose availability. Nature Communications, 9(1). 10.1038/s41467-018-04849-7 PMC6041281 29992949

[acel13431-bib-0079] Li, Y., Xu, M., Ding, X., Yan, C., Song, Z., Chen, L., Huang, X., Wang, X., Jian, Y., Tang, G., Tang, C., Di, Y., Mu, S., Liu, X., Liu, K., Li, T., Wang, Y., Miao, L., Guo, W., … Yang, C. (2016). Protein kinase C controls lysosome biogenesis independently of mTORC1. Nature Cell Biology, 18(10), 1065–1077. 10.1038/ncb3407 27617930

[acel13431-bib-0080] Liang, X. H., Jackson, S., Seaman, M., Brown, K., Kempkes, B., Hibshoosh, H., & Levine, B. (1999). Induction of autophagy and inhibition of tumorigenesis by beclin 1. Nature, 402(6762), 672–676. 10.1038/45257 10604474

[acel13431-bib-0081] Liao, Y., & Hung, M. C. (2010). Physiological regulation of Akt activity and stability. American Journal of Translational Research, 2(1), 19–42. 20182580 PMC2826820

[acel13431-bib-0084] Liu, P., Begley, M., Michowski, W., Inuzuka, H., Ginzberg, M., Gao, D., Tsou, P., Gan, W., Papa, A., Kim, B. M., Wan, L., Singh, A., Zhai, B., Yuan, M., Wang, Z., Gygi, S. P., Lee, T. H., Lu, K. P., Toker, A., … Wei, W. (2014). Cell‐cycle‐regulated activation of Akt kinase by phosphorylation at its carboxyl terminus. Nature, 508(7497), 541–545. 10.1038/nature13079 24670654 PMC4076493

[acel13431-bib-0082] Liu, J., Bi, X., Chen, T., Zhang, Q., Wang, S.‐X., Chiu, J.‐J., Liu, G.‐S., Zhang, Y., Bu, P., & Jiang, F. (2015). Shear stress regulates endothelial cell autophagy via redox regulation and Sirt1 expression. Cell Death & Disease, 6(7), e1827–e1827. 10.1038/cddis.2015.193 26181207 PMC4650738

[acel13431-bib-0083] Liu, L., & Cheng, Z. (2018). Forkhead Box O (FoxO) Transcription Factors in Autophagy, Metabolic Health, and Tissue Homeostasis. In: Turksen, K. (eds) Autophagy in Health and Disease. Stem Cell Biology and Regenerative Medicine. Humana Press, Cham. 10.1007/978-3-319-98146-8_4

[acel13431-bib-0085] Liu, P., Gan, W., Chin, Y. R., Ogura, K., Guo, J., Zhang, J., Wang, B., Blenis, J., Cantley, L. C., Toker, A., Su, B., & Wei, W. (2015). PtdIns(3,4,5)P3‐Dependent Activation of the mTORC2 Kinase Complex. Cancer Discovery, 5(11), 1194–1209. 10.1158/2159-8290.cd-15-0460 26293922 PMC4631654

[acel13431-bib-0086] Liu, P., Wang, Z., & Wei, W. (2014). Phosphorylation of Akt at the C‐terminal tail triggers Akt activation. Cell Cycle, 13(14), 2162–2164. 24933731 10.4161/cc.29584 PMC4111671

[acel13431-bib-0087] Liu, W., Wang, X., Liu, Z., Wang, Y., Yin, B., Yu, P., Duan, X., Liao, Z., Chen, Y., Liu, C., Li, X., Dai, Y., & Tao, Z. (2017). SGK1 inhibition induces autophagy‐dependent apoptosis via the mTOR‐Foxo3a pathway. British Journal of Cancer, 117(8), 1139–1153. 10.1038/bjc.2017.293 29017179 PMC5674106

[acel13431-bib-0088] Luo, Y., Xu, W., Li, G., & Cui, W. (2018). Weighing in on mTOR complex 2 signaling: The expanding role in cell metabolism. Oxidative Medicine and Cellular Longevity, 2018, 1–15. 10.1155/2018/7838647 PMC6232796 30510625

[acel13431-bib-0089] Macpherson, I. R., Rainero, E., Mitchell, L. E., van den Berghe, P. V. E., Speirs, C., Dozynkiewicz, M. A., Chaudhary, S., Kalna, G., Edwards, J., Timpson, P., & Norman, J. C. (2014). CLIC3 controls recycling of late endosomal MT1‐MMP and dictates invasion and metastasis in breast cancer. Journal of Cell Science, 127(Pt 18), 3893–3901. 10.1242/jcs.135947 25015290

[acel13431-bib-0090] Maestro, I., Boya, P., & Serum‐, M. A. (2020). and glucocorticoid‐induced kinase 1, a new therapeutic target for autophagy modulation in chronic diseases. Expert Opinion on Therapeutic Targets, 24(3), 231–243. 10.1080/14728222.2020.1730328 32067528

[acel13431-bib-0091] Mahoney, S. J., Narayan, S., Molz, L., Berstler, L. A., Kang, S. A., Vlasuk, G. P., & Saiah, E. (2018). A small molecule inhibitor of Rheb selectively targets mTORC1 signaling. Nature Communications, 9(1). 10.1038/s41467-018-03035-z PMC5803267 29416044

[acel13431-bib-0092] Mammucari, C., Milan, G., Romanello, V., Masiero, E., Rudolf, R., Del Piccolo, P., Burden, S. J., Di Lisi, R., Sandri, C., Zhao, J., Goldberg, A. L., Schiaffino, S., & Sandri, M. (2007). FoxO3 Controls Autophagy in Skeletal Muscle In Vivo. Cell Metabolism, 6(6), 458–471. 10.1016/j.cmet.2007.11.001 18054315

[acel13431-bib-0093] Manning, B. D., & Toker, A. (2017). AKT/PKB Signaling: Navigating the Network. Cell, 169(3), 381–405. 28431241 10.1016/j.cell.2017.04.001 PMC5546324

[acel13431-bib-0094] Marchand, B., Arsenault, D., Raymond‐Fleury, A., Boisvert, F. M., & Boucher, M. J. (2015). Glycogen synthase kinase‐3 (GSK3) inhibition induces prosurvival autophagic signals in human pancreatic cancer cells. Journal of Biological Chemistry, 290(9), 5592–5605. 10.1074/jbc.M114.616714 PMC4342473 25561726

[acel13431-bib-0095] Martina, J. A., Chen, Y., Gucek, M., & Puertollano, R. (2012). MTORC1 functions as a transcriptional regulator of autophagy by preventing nuclear transport of TFEB. Autophagy, 8(6), 903–914. 22576015 10.4161/auto.19653 PMC3427256

[acel13431-bib-0096] Martina, J. A., Diab, H. I., Lishu, L., Jeong‐A, L., Patange, S., Raben, N., & Puertollano, R. (2014). The Nutrient‐Responsive Transcription Factor TFE3 Promotes Autophagy, Lysosomal Biogenesis, and Clearance of Cellular Debris. Science Signaling, 7(309), ra9–ra9. 10.1126/scisignal.2004754 24448649 PMC4696865

[acel13431-bib-0097] Martina, J. A., & Puertollano, R. (2013). Rag GTPases mediate amino acid‐dependent recruitment of TFEB and MITF to lysosomes. Journal of Cell Biology, 200(4), 475–491. 10.1083/jcb.201209135 PMC3575543 23401004

[acel13431-bib-0098] Martinez‐Lopez, N., Athonvarangkul, D., & Singh, R. (2015). Autophagy and aging. Advances in Experimental Medicine and Biology, 2015(847), 73–87. 10.1007/978-1-4939-2404-2_3 PMC4644734 25916586

[acel13431-bib-0099] Martin‐Perez, M., Grillo, A. S., Ito, T. K., Valente, A. S., Han, J., Entwisle, S. W., Huang, H. Z., Kim, D., Yajima, M., Kaeberlein, M., & Villén, J. (2020). PKC downregulation upon rapamycin treatment attenuates mitochondrial disease. Nature Metabolism, 2(12), 1472–1481. 10.1038/s42255-020-00319-x PMC8017771 33324011

[acel13431-bib-0100] Mason, J. S., Wileman, T., & Chapman, T. (2018). Lifespan extension without fertility reduction following dietary addition of the autophagy activator Torin1 in Drosophila melanogaster. PLoS One, 13(1), e0190105. 29329306 10.1371/journal.pone.0190105 PMC5766080

[acel13431-bib-0101] Masui, K., Tanaka, K., Akhavan, D., Babic, I., Gini, B., Matsutani, T., Iwanami, A., Liu, F., Villa, G., Gu, Y., Campos, C., Zhu, S., Yang, H., Yong, W., Cloughesy, T., Mellinghoff, I., Cavenee, W., Shaw, R., & Mischel, P. (2013). mTOR Complex 2 Controls Glycolytic Metabolism in Glioblastoma through FoxO Acetylation and Upregulation of c‐Myc. Cell Metabolism, 18(5), 726–739. 10.1016/j.cmet.2013.09.013 24140020 PMC3840163

[acel13431-bib-0102] Mathew, R., Kongara, S., Beaudoin, B., Karp, C. M., Bray, K., Degenhardt, K., Chen, G., Jin, S., & White, E. (2007). Autophagy suppresses tumor progression by limiting chromosomal instability. Genes & Development, 21(11), 1367–1381. 10.1101/gad.1545107 17510285 PMC1877749

[acel13431-bib-0103] Medina, D. L., Di Paola, S., Peluso, I., Armani, A., De Stefani, D., Venditti, R., Montefusco, S., Scotto‐Rosato, A., Prezioso, C., Forrester, A., Settembre, C., Wang, W., Gao, Q., Xu, H., Sandri, M., Rizzuto, R., De Matteis, M. A., & Ballabio, A. (2015). Lysosomal calcium signalling regulates autophagy through calcineurin and TFEB. Nature Cell Biology, 17(3), 288–299. 10.1038/ncb3114 25720963 PMC4801004

[acel13431-bib-0104] Medina, D. L., Fraldi, A., Bouche, V., Annunziata, F., Mansueto, G., Spampanato, C., Puri, C., Pignata, A., Martina, J. A., Sardiello, M., Palmieri, M., Polishchuk, R., Puertollano, R., & Ballabio, A. (2011). Transcriptional Activation of Lysosomal Exocytosis Promotes Cellular Clearance. Developmental Cell, 21(3), 421–430. 10.1016/j.devcel.2011.07.016 21889421 PMC3173716

[acel13431-bib-0105] Mizunuma, M., Neumann‐Haefelin, E., Moroz, N., Li, Y., & Blackwell, T. K. (2014). mTORC2‐SGK‐1 acts in two environmentally responsive pathways with opposing effects on longevity. Aging Cell, 13(5), 869–878. 25040785 10.1111/acel.12248 PMC4172656

[acel13431-bib-0106] Mizushima, N., & Komatsu, M. (2011). Autophagy: renovation of cells and tissues. Cell, 147(4), 728–741. 22078875 10.1016/j.cell.2011.10.026

[acel13431-bib-0107] Monteiro, P., Rossé, C., Castro‐Castro, A., Irondelle, M., Lagoutte, E., Paul‐Gilloteaux, P., Desnos, C., Formstecher, E., Darchen, F., Perrais, D., Gautreau, A., Hertzog, M., & Chavrier, P. (2013). Endosomal WASH and exocyst complexes control exocytosis of MT1‐MMP at invadopodia. Journal of Cell Biology, 203(6), 1063–1079. 10.1083/jcb.201306162 PMC3871436 24344185

[acel13431-bib-0108] Mostowy, S. (2014). Multiple Roles of the Cytoskeleton in Bacterial Autophagy. PLoS Path, 10(11), e1004409. 10.1371/journal.ppat.1004409 PMC4239108 25412422

[acel13431-bib-0109] Murray, E. R., & Cameron, A. J. M. (2017). Towards specific inhibition of mTORC2. Aging, 9(12), 2461–2462. 10.18632/aging.101346 29232655 PMC5764381

[acel13431-bib-0110] Nojima, A., Yamashita, M., Yoshida, Y., Shimizu, I., Ichimiya, H., Kamimura, N., Kobayashi, Y., Ohta, S., Ishii, N., & Minamino, T. (2013). Haploinsufficiency of Akt1 Prolongs the Lifespan of Mice. PLoS One, 8(7), e69178. 10.1371/journal.pone.0069178 23935948 PMC3728301

[acel13431-bib-0111] Oh, W. J., & Jacinto, E. (2011). mTOR complex 2 signaling and functions. Cell Cycle, 10(14), 2305–2316. 10.4161/cc.10.14.16586 21670596 PMC3322468

[acel13431-bib-0112] Ozturk, D. G., Kocak, M., Akcay, A., Kinoglu, K., Kara, E., Buyuk, Y., Kazan, H., & Gozuacik, D. (2019). MITF‐MIR211 axis is a novel autophagy amplifier system during cellular stress. Autophagy, 15(3), 375–390. 10.1080/15548627.2018.1531197 30290719 PMC6351134

[acel13431-bib-0113] Palmieri, M., Impey, S., Kang, H., di Ronza, A., Pelz, C., Sardiello, M., & Ballabio, A. (2011). Characterization of the CLEAR network reveals an integrated control of cellular clearance pathways. Human Molecular Genetics, 20(19), 3852–3866. 10.1093/hmg/ddr306 21752829

[acel13431-bib-0114] Palmieri, M., Pal, R., Nelvagal, H. R., Lotfi, P., Stinnett, G. R., Seymour, M. L., Chaudhury, A., Bajaj, L., Bondar, V. V., Bremner, L., Saleem, U., Tse, D. Y., Sanagasetti, D., Wu, S. M., Neilson, J. R., Pereira, F. A., Pautler, R. G., Rodney, G. G., Cooper, J. D., & Sardiello, M. (2017). mTORC1‐independent TFEB activation via Akt inhibition promotes cellular clearance in neurodegenerative storage diseases. Nature Communications, 8, 14338. 10.1038/ncomms14338. Erratum in: Nat Commun. 2017 Jun 13;8:15793. PMID: 28165011; PMCID: PMC5303831. PMC5303831 28165011

[acel13431-bib-0115] Patergnani, S., Marchi, S., Rimessi, A., Bonora, M., Giorgi, C., Mehta, K. D., & Pinton, P. (2013). PRKCB/protein kinase C, beta and the mitochondrial axis as key regulators of autophagy. Autophagy, 9(9), 1367–1385. 10.4161/auto.25239 23778835

[acel13431-bib-0116] Pearce, L. R., Komander, D., & Alessi, D. R. (2010). The nuts and bolts of AGC protein kinases. Nature Reviews Molecular Cell Biology, 11(1), 9–22. 10.1038/nrm2822 20027184

[acel13431-bib-0117] Peña‐Llopis, S., Vega‐Rubin‐de‐Celis, S., Schwartz, J. C., Wolff, N. C., Tran, T. A. T., Zou, L., Xie, X.‐J., Corey, D. R., & Brugarolas, J. (2011). Regulation of TFEB and V‐ATPases by mTORC1. The EMBO Journal, 30(16), 3242–3258. 10.1038/emboj.2011.257 21804531 PMC3160667

[acel13431-bib-0118] Perera, R. M., & Zoncu, R. (2016). The Lysosome as a Regulatory Hub. Annual Review of Cell and Developmental Biology, 2016(32), 223–253. 10.1146/annurev-cellbio-111315-125125 PMC9345128 27501449

[acel13431-bib-0119] Peterson, T. R., Laplante, M., Thoreen, C. C., Sancak, Y., Kang, S. A., Kuehl, W. M., Gray, N. S., & Sabatini, D. M. (2009). DEPTOR Is an mTOR Inhibitor Frequently Overexpressed in Multiple Myeloma Cells and Required for Their Survival. Cell, 137(5), 873–886. 10.1016/j.cell.2009.03.046 19446321 PMC2758791

[acel13431-bib-0121] Polak, P., & Hall, M. N. (2006). mTORC2 Caught in a SINful Akt. Developmental Cell, 11(4), 433–434. 10.1016/j.devcel.2006.09.005 17011481

[acel13431-bib-0122] Pu, J., Guardia, C. M., Keren‐Kaplan, T., & Bonifacino, J. S. (2016). Mechanisms and functions of lysosome positioning. Journal of Cell Science, 129(23), 4329–4339. 27799357 10.1242/jcs.196287 PMC5201012

[acel13431-bib-0123] Raposo, G., & Marks, M. S. (2002). The dark side of lysosome‐related organelles: specialization of the endocytic pathway for melanosome biogenesis. Traffic, 3(4), 237–248. 10.1034/j.1600-0854.2002.030401.x 11929605

[acel13431-bib-0124] Risso, G., Blaustein, M., Pozzi, B., Mammi, P., & Srebrow, A. (2015). Akt/PKB: One kinase, many modifications. Biochemical Journal, 468(2), 203–214. 10.1042/BJ20150041 25997832

[acel13431-bib-0125] Roczniak‐Ferguson, A., Petit, C. S., Froehlich, F., Qian, S., Ky, J., Angarola, B., Walther, T. C., & Ferguson, S. M. (2012). The Transcription Factor TFEB Links mTORC1 Signaling to Transcriptional Control of Lysosome Homeostasis. Science Signaling, 5(228), ra42. 10.1126/scisignal.2002790 22692423 PMC3437338

[acel13431-bib-0126] Romeo, Y., Moreau, J., Zindy, P.‐J., Saba‐El‐Leil, M., Lavoie, G., Dandachi, F., Baptissart, M., Borden, K. L. B., Meloche, S., & Roux, P. P. (2013). RSK regulates activated BRAF signalling to mTORC1 and promotes melanoma growth. Oncogene, 32(24), 2917–2926. 10.1038/onc.2012.312 22797077 PMC4440665

[acel13431-bib-0127] Rubinsztein, D. C., Marino, G., & Kroemer, G. (2011). Autophagy and aging. Cell, 146(5), 682–695. 21884931 10.1016/j.cell.2011.07.030

[acel13431-bib-0128] Salo, J., Lehenkari, P., Mulari, M., Metsikko, K., & Vaananen, H. K. (1997). Removal of osteoclast bone resorption products by transcytosis. Science, 276(5310), 270–273. 9092479 10.1126/science.276.5310.270

[acel13431-bib-0129] Sancak, Y., Peterson, T. R., Shaul, Y. D., Lindquist, R. A., Thoreen, C. C., Bar‐Peled, L., & Sabatini, D. M. (2008). The Rag GTPases Bind Raptor and Mediate Amino Acid Signaling to mTORC1. Science, 320(5882), 1496–1501. 10.1126/science.1157535 18497260 PMC2475333

[acel13431-bib-0130] Sancak, Y., Thoreen, C. C., Peterson, T. R., Lindquist, R. A., Kang, S. A., Spooner, E., Carr, S. A., & Sabatini, D. M. (2007). PRAS40 Is an Insulin‐Regulated Inhibitor of the mTORC1 Protein Kinase. Molecular Cell, 25(6), 903–915. 10.1016/j.molcel.2007.03.003 17386266

[acel13431-bib-0131] Sarbassov, D. D., Ali, S. M., Sengupta, S., Sheen, J.‐H., Hsu, P. P., Bagley, A. F., Markhard, A. L., & Sabatini, D. M. (2006). Prolonged Rapamycin Treatment Inhibits mTORC2 Assembly and Akt/PKB. Molecular Cell, 22(2), 159–168. 10.1016/j.molcel.2006.03.029 16603397

[acel13431-bib-0132] Sarbassov, D. D., Guertin, D. A., Ali, S. M., & Sabatini, D. M. (2005). Phosphorylation and regulation of Akt/PKB by the rictor‐mTOR complex. Science, 307(5712), 1098–1101. 15718470 10.1126/science.1106148

[acel13431-bib-0133] Saxton, R. A., & Sabatini, D. M. (2017). mTOR Signaling in Growth, Metabolism, and Disease. Cell, 168(6), 960–976. 10.1016/j.cell.2017.02.004 28283069 PMC5394987

[acel13431-bib-0134] Schneider, J. L., Suh, Y., & Cuervo, A. M. (2014). Deficient chaperone‐mediated autophagy in liver leads to metabolic dysregulation. Cell Metabolism, 20(3), 417–432. 10.1016/j.cmet.2014.06.009 25043815 PMC4156578

[acel13431-bib-0135] Schreiber, K. H., Arriola Apelo, S. I., Yu, D., Brinkman, J. A., Velarde, M. C., Syed, F. A., Liao, C.‐Y., Baar, E. L., Carbajal, K. A., Sherman, D. S., Ortiz, D., Brunauer, R., Yang, S. E., Tzannis, S. T., Kennedy, B. K., & Lamming, D. W. (2019). A novel rapamycin analog is highly selective for mTORC1 in vivo. Nature Communications, 10(1), 3194. 10.1038/s41467-019-11174-0 PMC6642166 31324799

[acel13431-bib-0136] Schuck, S. (2020). Microautophagy – distinct molecular mechanisms handle cargoes of many sizes. Journal of Cell Science, 133(17), jcs246322. 32907930 10.1242/jcs.246322

[acel13431-bib-0137] Settembre, C., Zoncu, R., Medina, D. L., Vetrini, F., Erdin, S., Erdin, S., Huynh, T., Ferron, M., Karsenty, G., Vellard, M. C., Facchinetti, V., Sabatini, D. M., & Ballabio, A. (2012). A lysosome‐to‐nucleus signalling mechanism senses and regulates the lysosome via mTOR and TFEB. The EMBO Journal, 31(5), 1095–1108. 10.1038/emboj.2012.32 22343943 PMC3298007

[acel13431-bib-0138] Shen, M., Jiang, Y., Guan, Z., Cao, Y., Li, L., Liu, H., & Sun, S. (2017). Protective mechanism of FSH against oxidative damage in mouse ovarian granulosa cells by repressing autophagy. Autophagy, 13(8), 1364–1385. 10.1080/15548627.2017.1327941 28598230 PMC5584866

[acel13431-bib-0139] Soukas, A. A., Kane, E. A., Carr, C. E., Melo, J. A., & Ruvkun, G. (2009). Rictor/TORC2 regulates fat metabolism, feeding, growth, and life span in Caenorhabditis elegans. Genes & Development, 23(4), 496–511. 10.1101/gad.1775409 19240135 PMC2648650

[acel13431-bib-0140] Srivastava, R. K., Li, C., Khan, J., Banerjee, N. S., Chow, L. T., & Athar, M. (2019). Combined mTORC1/mTORC2 inhibition blocks growth and induces catastrophic macropinocytosis in cancer cells. Proceedings of the National Academy of Sciences, 116(49), 24583–24592. 10.1073/pnas.1911393116 PMC6900636 31732667

[acel13431-bib-0141] Steffan, J. J., Dykes, S. S., Coleman, D. T., Adams, L. K., Rogers, D., Carroll, J. L., Williams, B. J., & Cardelli, J. A. (2014). Supporting a Role for the GTPase Rab7 in Prostate Cancer Progression. PLoS One, 9(2), e87882. 10.1371/journal.pone.0087882 24505328 PMC3914878

[acel13431-bib-0142] Swiatkowski, P., Nikolaeva, I., Kumar, G., Zucco, A., Akum, B. F., Patel, M. V., D’Arcangelo, G., & Firestein, B. L. (2017). Role of Akt‐independent mTORC1 and GSK3β signaling in sublethal NMDA‐induced injury and the recovery of neuronal electrophysiology and survival. Scientific Reports, 7(1). 10.1038/s41598-017-01826-w PMC5431483 28484273

[acel13431-bib-0143] Takamura, A., Komatsu, M., Hara, T., Sakamoto, A., Kishi, C., Waguri, S., Eishi, Y., Hino, O., Tanaka, K., & Mizushima, N. (2011). Autophagy‐deficient mice develop multiple liver tumors. Genes & Development, 25(8), 795–800. 10.1101/gad.2016211 21498569 PMC3078705

[acel13431-bib-0144] Tatebe, H., Murayama, S., Yonekura, T., Hatano, T., Richter, D., Furuya, T., Kataoka, S., Furuita, K., Kojima, C., & Shiozaki, K. (2017). Substrate specificity of TOR complex 2 is determined by a ubiquitin‐fold domain of the Sin1 subunit. eLife, 6. 10.7554/elife.19594 PMC5340527 28264193

[acel13431-bib-0145] Tato, I., Bartrons, R., Ventura, F., & Rosa, J. L. (2011). Amino acids activate mammalian target of rapamycin complex 2 (mTORC2) via PI3K/Akt signaling. Journal of Biological Chemistry, 286(8), 6128–6142. 10.1074/jbc.M110.166991 PMC3057817 21131356

[acel13431-bib-0146] Thoreen, C. C., Kang, S. A., Chang, J. W., Liu, Q., Zhang, J., Gao, Y. I., Reichling, L. J., Sim, T., Sabatini, D. M., & Gray, N. S. (2009). An ATP‐competitive Mammalian Target of Rapamycin Inhibitor Reveals Rapamycin‐resistant Functions of mTORC1. Journal of Biological Chemistry, 284(12), 8023–8032. 10.1074/jbc.M900301200 PMC2658096 19150980

[acel13431-bib-0147] Thoreen, C. C., & Sabatini, D. M. (2009). Rapamycin inhibits mTORC1, but not completely. Autophagy, 5(5), 725–726. 19395872 10.4161/auto.5.5.8504

[acel13431-bib-0148] Tobita, T., Guzman‐Lepe, J., Takeishi, K., Nakao, T., Wang, Y., Meng, F., Deng, C.‐X., Collin de l’Hortet, A., & Soto‐Gutierrez, A. (2016). SIRT1 Disruption in Human Fetal Hepatocytes Leads to Increased Accumulation of Glucose and Lipids. PLoS One, 11(2), e0149344. 10.1371/journal.pone.0149344 26890260 PMC4758736

[acel13431-bib-0149] Tulsiani, D. R., Abou‐Haila, A., Loeser, C. R., & Pereira, B. M. (1998). The biological and functional significance of the sperm acrosome and acrosomal enzymes in mammalian fertilization. Experimental Cell Research, 240(2), 151–164. 10.1006/excr.1998.3943 9596988

[acel13431-bib-0150] van der Vos, K. E., Eliasson, P., Proikas‐Cezanne, T., Vervoort, S. J., van Boxtel, R., Putker, M., van Zutphen, I. J., Mauthe, M., Zellmer, S., Pals, C., Verhagen, L. P., Groot Koerkamp, M. J. A., Braat, A. K., Dansen, T. B., Holstege, F. C., Gebhardt, R., Burgering, B. M., & Coffer, P. J. (2012). Modulation of glutamine metabolism by the PI(3)K–PKB–FOXO network regulates autophagy. Nature Cell Biology, 14(8), 829–837. 10.1038/ncb2536 22820375

[acel13431-bib-0151] Vlahakis, A., Graef, M., Nunnari, J., & Powers, T. (2014). TOR complex 2‐Ypk1 signaling is an essential positive regulator of the general amino acid control response and autophagy. Proceedings of the National Academy of Sciences, 111(29), 10586–10591. 10.1073/pnas.1406305111 PMC4115538 25002487

[acel13431-bib-0152] Wang, F., Xu, C., Reece, E. A., Li, X., Wu, Y., Harman, C., Yu, J., Dong, D., Wang, C., Yang, P., Zhong, J., & Yang, P. (2017). Protein kinase C‐alpha suppresses autophagy and induces neural tube defects via miR‐129‐2 in diabetic pregnancy. Nature Communications, 8(1), 15182. 10.1038/ncomms15182 PMC5424165 28474670

[acel13431-bib-0153] Wang, J., Whiteman, M. W., Lian, H., Wang, G., Singh, A., Huang, D., & Denmark, T. (2009). A Non‐canonical MEK/ERK Signaling Pathway Regulates Autophagy via Regulating Beclin 1. Journal of Biological Chemistry, 284(32), 21412–21424. 10.1074/jbc.m109.026013 PMC2755866 19520853

[acel13431-bib-0154] Wang, R. H., Kim, H. S., Xiao, C., Xu, X., Gavrilova, O., & Deng, C. X. (2011). Hepatic Sirt1 deficiency in mice impairs mTorc2/Akt signaling and results in hyperglycemia, oxidative damage, and insulin resistance. Journal of Clinical Investigation, 121(11), 4477–4490. 10.1172/JCI46243 PMC3204833 21965330

[acel13431-bib-0155] Wang, R. C., Wei, Y., An, Z., Zou, Z., Xiao, G., Bhagat, G., White, M., Reichelt, J., & Levine, B. (2012). Akt‐Mediated Regulation of Autophagy and Tumorigenesis Through Beclin 1 Phosphorylation. Science, 338(6109), 956–959. 10.1126/science.1225967 23112296 PMC3507442

[acel13431-bib-0156] Wang, S., & Gu, K. (2018). Insulin‐like growth factor 1 inhibits autophagy of human colorectal carcinoma drug‐resistant cells via the protein kinase B/mammalian target of rapamycin signaling pathway. Molecular Medicine Reports, 17(2), 2952–2956. 29257307 10.3892/mmr.2017.8272 PMC5783513

[acel13431-bib-0157] Wang, Y., Xu, W., Yan, Z., Zhao, W., Mi, J., Li, J., & Yan, H. (2018). Metformin induces autophagy and G0/G1 phase cell cycle arrest in myeloma by targeting the AMPK/mTORC1 and mTORC2 pathways. Journal of Experimental & Clinical Cancer Research, 37(1). 10.1186/s13046-018-0731-5 PMC5859411 29554968

[acel13431-bib-0158] Warr, M. R., Binnewies, M., Flach, J., Reynaud, D., Garg, T., Malhotra, R., Debnath, J., & Passegué, E. (2013). FOXO3A directs a protective autophagy program in haematopoietic stem cells. Nature, 494(7437), 323–327. 10.1038/nature11895 23389440 PMC3579002

[acel13431-bib-0159] Webb, A. E., & Brunet, A. (2014). FOXO transcription factors: Key regulators of cellular quality control. Trends in Biochemical Sciences, 39(4), 159–169. 10.1016/j.tibs.2014.02.003 24630600 PMC4021867

[acel13431-bib-0160] Wen, H., Zhan, L., Chen, S., Long, L., & Xu, E. (2017). Rab7 may be a novel therapeutic target for neurologic diseases as a key regulator in autophagy. Journal of Neuroscience Research, 95(10), 1993–2004. 28186670 10.1002/jnr.24034

[acel13431-bib-0161] White, E., & DiPaola, R. S. (2009). The double‐edged sword of autophagy modulation in cancer. Clinical Cancer Research, 15(17), 5308–5316. 10.1158/1078-0432.CCR-07-5023 19706824 PMC2737083

[acel13431-bib-0162] Wong, V. K. W., Zeng, W., Chen, J., Yao, X. J., Leung, E. L. H., Wang, Q. Q., Chiu, P., Ko, B. C. B., & Law, B. Y. K. (2017). Tetrandrine, an Activator of Autophagy, Induces Autophagic Cell Death via PKC‐α Inhibition and mTOR‐Dependent Mechanisms. Frontiers in Pharmacology, 8. 10.3389/fphar.2017.00351 PMC5462963 28642707

[acel13431-bib-0163] Xie, J., Wang, X., & Proud, C. G. (2018). Who does TORC2 talk to. Biochemical Journal, 475(10), 1721–1738. 10.1042/BCJ20180130 29794170

[acel13431-bib-0164] Xiong, J. (2015). Atg7 in development and disease: panacea or Pandora’s Box? Protein and Cell, 6(10), 722–734. 26404030 10.1007/s13238-015-0195-8 PMC4598325

[acel13431-bib-0165] Xiong, Y., Yepuri, G., Forbiteh, M., Yu, Y. I., Montani, J.‐P., Yang, Z., & Ming, X.‐F. (2014). ARG2 impairs endothelial autophagy through regulation of MTOR and PRKAA/AMPK signaling in advanced atherosclerosis. Autophagy, 10(12), 2223–2238. 10.4161/15548627.2014.981789 25484082 PMC4502672

[acel13431-bib-0166] Xue, G., Kohler, R., Tang, F., Hynx, D., Wang, Y., Orso, F., Prêtre, V., Ritschard, R., Hirschmann, P., Cron, P., Roloff, T., Dummer, R., Mandalà, M., Bichet, S., Genoud, C., Meyer, A. G., Muraro, M. G., Spagnoli, G. C., Taverna, D., … Wicki, A. (2017). mTORC1/autophagy‐regulated MerTK in mutant BRAFV600 melanoma with acquired resistance to BRAF inhibition. Oncotarget, 8(41), 69204–69218. 10.18632/oncotarget.18213 29050198 PMC5642473

[acel13431-bib-0167] Yang, G., Murashige, D. S., Humphrey, S. J., & James, D. E. (2015). A Positive Feedback Loop between Akt and mTORC2 via SIN1 Phosphorylation. Cell Reports, 12(6), 937–943. 10.1016/j.celrep.2015.07.016 26235620

[acel13431-bib-0168] Yang, Q., Inoki, K., Ikenoue, T., & Guan, K. L. (2006). Identification of Sin1 as an essential TORC2 component required for complex formation and kinase activity. Genes & Development, 20(20), 2820–2832. 17043309 10.1101/gad.1461206 PMC1619946

[acel13431-bib-0169] Yang, S., Wang, X., Contino, G., Liesa, M., Sahin, E., Ying, H., Bause, A., Li, Y., Stommel, J. M., Dell'Antonio, G., Mautner, J., Tonon, G., Haigis, M., Shirihai, O. S., Doglioni, C., Bardeesy, N., & Kimmelman, A. C. (2011). Pancreatic cancers require autophagy for tumor growth. Genes & Development, 25(7), 717–729. 10.1101/gad.2016111 21406549 PMC3070934

[acel13431-bib-0170] Yu, D., Tomasiewicz, J. L., Yang, S. E., Miller, B. R., Wakai, M. H., Sherman, D. S., Cummings, N. E., Baar, E. L., Brinkman, J. A., Syed, F. A., & Lamming, D. W. (2019). Calorie‐Restriction‐Induced Insulin Sensitivity Is Mediated by Adipose mTORC2 and Not Required for Lifespan Extension. Cell Reports, 29(1), 236–248.e3. 10.1016/j.celrep.2019.08.084 31577953 PMC6820997

[acel13431-bib-0171] Yu, X., & Long, Y. C. (2016). Crosstalk between cystine and glutathione is critical for the regulation of amino acid signaling pathways and ferroptosis. Scientific Reports, 6, 30033. 10.1038/srep30033 27425006 PMC4948025

[acel13431-bib-0172] Yu, Y., Yang, L., Han, S., Wu, Y., Liu, L., Chang, Y., Wang, X., & Chai, J. (2019). MIR‐190B Alleviates Cell Autophagy and Burn‐Induced Skeletal Muscle Wasting via Modulating PHLPP1/Akt/FoxO3A Signaling Pathway. Shock, 52(5), 513–521. 10.1097/SHK.0000000000001284 30407372

[acel13431-bib-0173] Yuan, H. X., & Guan, K. L. (2015). The SIN1‐PH domain connects mTORC2 to PI3K. Cancer Discovery, 5(11), 1127–1129. 26526694 10.1158/2159-8290.CD-15-1125 PMC4638136

[acel13431-bib-0174] Zhang, C., & Cuervo, A. M. (2008). Restoration of chaperone‐mediated autophagy in aging liver improves cellular maintenance and hepatic function. Nature Medicine, 14(9), 959–965. 10.1038/nm.1851 PMC2722716 18690243

[acel13431-bib-0175] Zhang, J., Xu, K., Liu, P., Geng, Y., Wang, B., Gan, W., Guo, J., Wu, F., Chin, Y., Berrios, C., Lien, E., Toker, A., DeCaprio, J., Sicinski, P., & Wei, W. (2016). Inhibition of Rb Phosphorylation Leads to mTORC2‐Mediated Activation of Akt. Molecular Cell, 62(6), 929–942. 10.1016/j.molcel.2016.04.023 27237051 PMC4912424

[acel13431-bib-0176] Zhang, T., Zhou, Q., Ogmundsdottir, M. H., Möller, K., Siddaway, R., Larue, L., Hsing, M., Kong, S. W., Goding, C., Palsson, A., Steingrimsson, E., & Pignoni, F. (2015). Mitf is a master regulator of the v‐ATPase forming an Mitf/v‐ATPase/TORC1 control module for cellular homeostasis. Journal of Cell Science. 10.1242/jcs.173807 PMC4540953 26092939

[acel13431-bib-0177] Zhang, Y., Swanda, R. V., Nie, L., Liu, X., Wang, C., Lee, H., Lei, G., Mao, C., Koppula, P., Cheng, W., Zhang, J., Xiao, Z., Zhuang, L. I., Fang, B., Chen, J., Qian, S.‐B., & Gan, B. (2021). mTORC1 couples cyst(e)ine availability with GPX4 protein synthesis and ferroptosis regulation. Nature Communications, 12(1). 1589. 10.1038/s41467-021-21841-w PMC7952727 33707434

[acel13431-bib-0178] Zhao, J., Brault, J. J., Schild, A., Cao, P., Sandri, M., Schiaffino, S., Lecker, S. H., & Goldberg, A. L. (2007). FoxO3 Coordinately Activates Protein Degradation by the Autophagic/Lysosomal and Proteasomal Pathways in Atrophying Muscle Cells. Cell Metabolism, 6(6), 472–483. 10.1016/j.cmet.2007.11.004 18054316

[acel13431-bib-0179] Zhao, Y., Yang, J., Liao, W., Liu, X., Zhang, H., Wang, S., Wang, D., Feng, J., Yu, L., & Zhu, W.‐G. (2010). Cytosolic FoxO1 is essential for the induction of autophagy and tumour suppressor activity. Nature Cell Biology, 12(7), 665–675. 10.1038/ncb2069 20543840

[acel13431-bib-0180] Zhou, B., Kreuzer, J., Kumsta, C., Wu, L., Kamer, K. J., Cedillo, L., Zhang, Y., Li, S., Kacergis, M. C., Webster, C. M., Fejes‐Toth, G., Naray‐Fejes‐Toth, A., Das, S., Hansen, M., Haas, W., & Soukas, A. A. (2019). Mitochondrial Permeability Uncouples Elevated Autophagy and Lifespan Extension. Cell, 177(2), 299–314. 10.1016/j.cell.2019.02.013 30929899 PMC6610881

[acel13431-bib-0181] Zinzalla, V., Stracka, D., Oppliger, W., & Hall, M. N. (2011). Activation of mTORC2 by association with the ribosome. Cell, 144(5), 757–768. 10.1016/j.cell.2011.02.014 21376236

[acel13431-bib-0182] Zoncu, R., Efeyan, A., & Sabatini, D. M. (2011). mTOR: from growth signal integration to cancer, diabetes and ageing. Nature Review Molecular Cell Biology, 12(1), 21–35. 21157483 10.1038/nrm3025 PMC3390257

[acel13431-bib-0183] Zuleger, T., Heinzelbecker, J., Takacs, Z., Hunter, C., Voelkl, J., Lang, F., & Proikas‐Cezanne, T. (2018). SGK1 Inhibits Autophagy in Murine Muscle Tissue. Oxidative Medicine and Cellular Longevity, 2018, 1–12. 10.1155/2018/4043726 PMC5937381 29849891

